# Case Report: Familial hypocalciuric hypercalcemia type 1 with a novel mutation combined with Gitelman syndrome and a review of the literature

**DOI:** 10.3389/fendo.2025.1503128

**Published:** 2025-02-25

**Authors:** Taoyuan He, Xinyu Li, Guosheng Li, Wanyang Wang, Hongjun Fu, Zhengnan Gao, Xuhan Liu

**Affiliations:** ^1^ Department of Endocrinology, Central Hospital of Dalian University of Technology, Dalian, China; ^2^ Graduate School, Dalian Medical University, Dalian, China; ^3^ Laboratory Pathology Department, Ningbo Clinical Pathology Diagnosis Center, Ningbo, China; ^4^ Tianjin Medical Laboratory, Beijing Genomics Institute (BGI)-Tianjin, BGI-Shenzhen, Tianjin, China

**Keywords:** familial hypocalciuric hypercalcemia, familial hypocalciuric hypercalcemia type 1, Gitelman syndrome, chondrocalcinosis, diabetes mellitus

## Abstract

**Introduction:**

Familial hypocalciuric hypercalcemia (FHH) is an autosomal dominant disorder caused by an inactivating mutation in the *CASR* gene, while Gitelman syndrome (GS) is an autosomal recessive renal tubular disorder resulting from a pathogenic mutation in the *SLC12A3* gene. Both genetic disorders are relatively rare. This report presents a patient with both FHH and GS, exhibiting unique clinical and genetic complexities.

**Case summary:**

We report a case of a 69-year-old Asian female patient who had previously presented to the hospital on multiple occasions with complaints of joint stiffness, fatigue, dizziness, or other symptoms. The patient was readmitted to the hospital at the age of 66, presenting with the following clinical findings: hypocalciuria, hypercalcemia, normal or mildly elevated parathyroid hormone (PTH) levels, hypokalemia, hypomagnesemia, hypophosphatemia, normal blood pressure, chondrocalcinosis (CC), and diabetes mellitus. Our careful analysis suggested that the patient might have the co-occurrence of GS and FHH. Genetic testing revealed a novel heterozygous *CASR* p.Tyr161* mutation and a homozygous *SLC12A3* p.Thr60Met mutation, which ultimately confirmed the diagnosis of familial hypocalciuric hypercalcemia type 1 (FHH1) combined with GS.

**Conclusion:**

For the first time, we report a case of FHH combined with GS. The novel *CASR* mutation in this patient expands the variant spectrum of FHH, provides new genetic evidence for its pathogenesis, and underscores the importance of genetic counseling for consanguineous families. This case also suggests a potential association between FHH and CC, the mechanism of which warrants further investigation. In addition, this report highlights possible potential interactions between FHH and GS. Clinically, hypokalemia and hypomagnesemia associated with GS are more detrimental than hypercalcemia linked to FHH and should be prioritized in management. Finally, genetic testing and molecular diagnostics are crucial for pediatric and adolescent populations with FHH and/or GS, and further studies are needed to clarify the genotypic and phenotypic relationships between FHH and GS comorbidities.

## Introduction

Familial hypocalciuric hypercalcemia (FHH) is an autosomal dominant disorder, familial hypocalciuric hypercalcemia type 1 (FHH1) is the most prevalent form of FHH and is caused by an inactivating mutation in the *CASR* gene, resulting in a loss of function of the calcium-sensing receptor (CaSR). FHH exhibits a similar phenotype across its various subtypes. It typically presents with a triad of symptoms: lifelong non-progressive hypercalcemia, normal or mildly elevated parathyroid hormone (PTH) levels, and hypocalciuria. Hypophosphatemia is commonly observed, and mild hypermagnesemia may also occur (1, 2). Gitelman syndrome (GS) is an autosomal recessive renal tubular disorder caused by a pathogenic variant in the *SLC12A3*, which encodes the thiazide-sensitive Na-Cl cotransporter (NCC). NCC mediates sodium and chloride reabsorption in the distal convoluted tubule (DCT). GS is clinically characterized by hypokalemia, hypomagnesemia, hypocalciuria, hypochloremic metabolic alkalosis, hyperreninemia, and secondary hyperaldosteronism. Patients typically exhibit normal or low blood pressure (3–5).

We present a Chinese female patient with a rare combination of GS and FHH. Clinically, the patient presented with hypocalciuria, hypercalcemia, normal or mildly elevated PTH levels, hypokalemia, hypomagnesemia, hypophosphatemia, normal blood pressure, chondrocalcinosis (CC), and diabetes mellitus. A novel heterozygous mutation in *CASR*, NM_000388.3:c.483delCinsGA (p.Tyr161*), is detected in this patient. This mutation has not been reported in variant databases including: GnomAD, 1000 Genome, ClinVar and HGMD. The novel *CASR* mutations expands the variant spectrum of FHH, broadening the range of known *CASR* variants associated with the condition, and underscores the importance of genetic counseling for offspring of consanguineous marriages. This report also suggests a potential association between FHH and CC, while exploring the possible mechanisms of interaction between FHH and GS. Clinically, patients with co-occurring FHH and GS require individualized treatment strategies to prevent complications. Finally, this case underscores the importance of genetic testing and molecular diagnosis in pediatric and adolescent populations with FHH and/or GS. Further studies involving multiple pedigrees, multicenter research, and molecular mechanisms are crucial to elucidating the genotypic and phenotypic relationships between FHH and GS comorbidities.

## Case presentation

A 69-year-old woman presented to our department with a 29-year history of bilateral elbow stiffness, which had worsened over the past three months, accompanied by limb soreness. At 44, she developed bilateral knee stiffness; subsequent hospital examinations revealed meniscal calcification in both knees, but she declined treatment. At age 50, she developed fatigue and was diagnosed with elevated blood calcium levels. She was admitted to Peking Union Medical College Hospital, where tests revealed slightly elevated PTH levels and hypocalciuria, but no abnormalities were observed in the 99Tc-sestamibi (MIBI) scintigraphy. Despite recommendations for further hospitalization for detailed investigations, she declined. Subsequently, the patient experienced limited elbow extension and joint calcification. At the age of 51, she was diagnosed with type 2 diabetes. In 2014, at the age of 59, she was treated for dizziness in the neurology department of our hospital. Subsequent examinations revealed hypercalcemia, hypokalemia, hypomagnesemia, and hypophosphatemia. She was referred to the endocrinology department for further treatment. A case discussion was held, suggesting a high likelihood of FHH, but she declined genetic testing.

She denies any history of hypertension, coronary heart disease, hepatitis, tuberculosis, drug allergies, or surgeries. Originally from Lanzhou City, Gansu Province, she currently resides in Dalian City, Liaoning Province. She has never smoked or consumed alcohol. Menstruation commenced at 15 and ceased at 41 without dysmenorrhea. She married at 28 and has a daughter. The patient’s parents were consanguineous (cousins). The patient denied any family history of hypertension, tumors, or related conditions. The patient’s body temperature was 36.2°C, pulse rate was 74 beats per minute, and blood pressure was 135/80 mmHg. The respiratory rate registered at 18 breaths per minute. The patient weighs 40 kg and is 155 cm tall, with a waist circumference of 77 cm and a Body Mass Index (BMI) of 16.6 kg/m².

Laboratory Tests(For key data, refer to **Table 1**; additional laboratory test results are available in **Supplementary Table 1**): The patient exhibited hypercalcemia, hypocalciuria, hypokalemia, hypomagnesemia and hypophosphatemia, low or deficient levels of 25-hydroxyvitamin D, and normal or mildly elevated PTH levels. The patient did not exhibit hypochloremic metabolic alkalosis and renin-angiotensin-aldosterone system (RAAS) activation(The blood pH was 7.404, arterial partial pressure of carbon dioxide (PaCO2) at 44.0 mmHg, actual bicarbonate concentration at 26.9 mmol/L, standard bicarbonate concentration at 26.0 mmol/L, buffer base at 1.8 mmol/L, renin at 19 pg/mL, angiotensin II at 40 pg/mL, and aldosterone at 130 pg/mL). The patient’s calcium/creatinine clearance ratios (CCCR) were 0.004 and 0.003, both below 0.01.

**Table 1:** Laboratory data.

Variables	April 2014	January 2021	Normal range
Serum Calcium(mmol/L)	3.04	3.28	2.10-2.60
Serum Phosphorus(mmol/L)	0.75	0.78	0.80-1.05
Serum Potassium(mmol/L)	2.80	3.30	3.50-5.30
Serum Magnesium(mmol/L)	0.39	0.36	0.66-1.07
Serum Sodium(mmol/L)	139.2	133.7	137.0-147.0
Serum Chloride(mmol/L)	97.6	96.5	99.0-110.0
Serum Creatinine (umol/L)	34	26	44-115
PTH levels (pg/mL)	58.2	89.9	18.5-88.0
25(OH) Vitamin D(ng/mL)	21.7	19.8	>30
24-hour Urine Volume(L)	2.10	2.30	
24-hour Urine Phosphorus(mmol/24h)	9.3	24.5	22.0-48.0
24-hour Urine Creatinine(mmol/24h)	3.5	5.95	7.0-18.0
24-hour Urine Calcium(mmol/24h)	1.37	1.93	2.50-7.50
24-hour Urine Potassium(mmol/24h)	27.8	52.7	25.0-100.0
24-hour Urine Sodium(mmol/24h)	127.5	154.3	130.0-260.0
24-hour Urine Chloride(mmol/24h)	113.8	159.6	170.0-255.0
CCCR	0.004	0.003	≥0.01

CCCR, calcium/creatinine clearance ratio, CCCR: <0.01 suggests FHH and ≥0.01 suggests PHPT. The normal range refers to the standard values established by our hospital’s laboratory.

**Figure 1:**
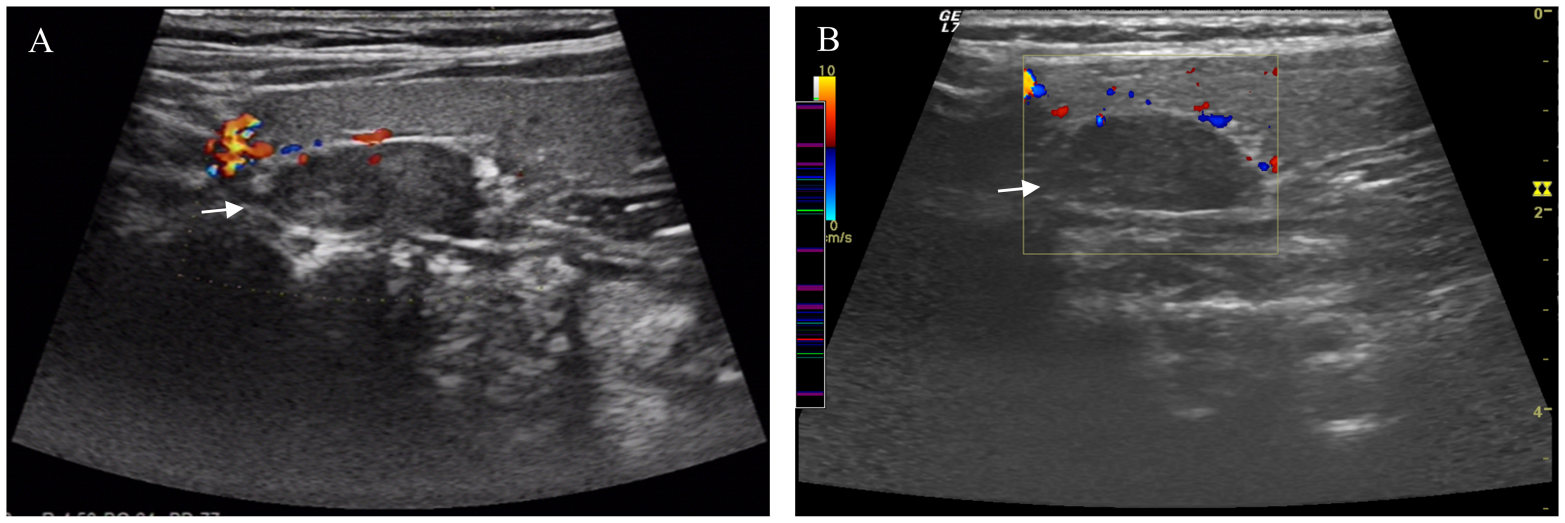
Ultrasound Imaging of the Parathyroid Glands. A hypoechoic nodule was observed on the left side of the parathyroid gland in the 2014 ultrasound **(A)**. The same nodule showed no significant changes in the 2021 ultrasound **(B)**. Arrow pointing to parathyroid nodule.

Normal thyroid function (free triiodothyronine (FT3), free thyroxine (FT4), thyroid-stimulating hormone (TSH), and anti-thyroid peroxidase antibodies) was observed, with slightly elevated anti-thyroglobulin antibodies (71.82 U/mL). Complete blood count, liver function (alkaline phosphatase: 48 U/L), and kidney function (urea: 2.71 mmol/L, creatinine: 34 µmol/L) were approximately normal. Serum aldosterone levels were within normal range. Growth hormone, follicle-stimulating hormone (FSH), luteinizing hormone (LH), estradiol (E2), progesterone (P4), prolactin (PRL), testosterone, and cortisol levels were within normal ranges. Additionally, serum tumor markers, including alpha-fetoprotein, carcinoembryonic antigen, and carbohydrate antigen 19-9, as well as carbohydrate antigen 12-5 and 15-3, were also found to be normal. Serum kappa light chains and lambda light chains were within normal limits. The urine pH was 7.0, and the specific gravity was consistently measured at 1.010 across three samples. Blood osmolality was recorded at 288 mOsm/kg of water, while the osmolality in random urine was 849 mOsm/kg of water. Imaging and Additional Examinations: Ultrasound of the Parathyroid Glands: A hypoechoic nodule was identified on the upper pole of the left thyroid lobe, dorsal to the upper pole, suggestive of potential parathyroid lesions. This nodule showed no significant change over a seven-year period (**Figure 1**).

99Tcm- MIBI scintigraphy of the parathyroid glands:No abnormalities were detected in the parathyroid glands. Chest CT(Computerized Tomography) Scan: Bilateral shoulder joint changes were noted, characterized by irregular morphology and uneven density; no other significant abnormalities were observed. Head CT Scan: Showed no significant abnormalities; however, multiple punctate calcifications were present bilaterally in the eyes. Ultrasound Examination: Liver, gallbladder, spleen, pancreas, urinary system, gynecological, and breast examinations revealed no significant abnormalities. Gastroenteroscopy: Revealed no abnormalities. Bone densitometry indicates decreased bone mass in the right femur and lumbar vertebrae.

**Figure 2:**
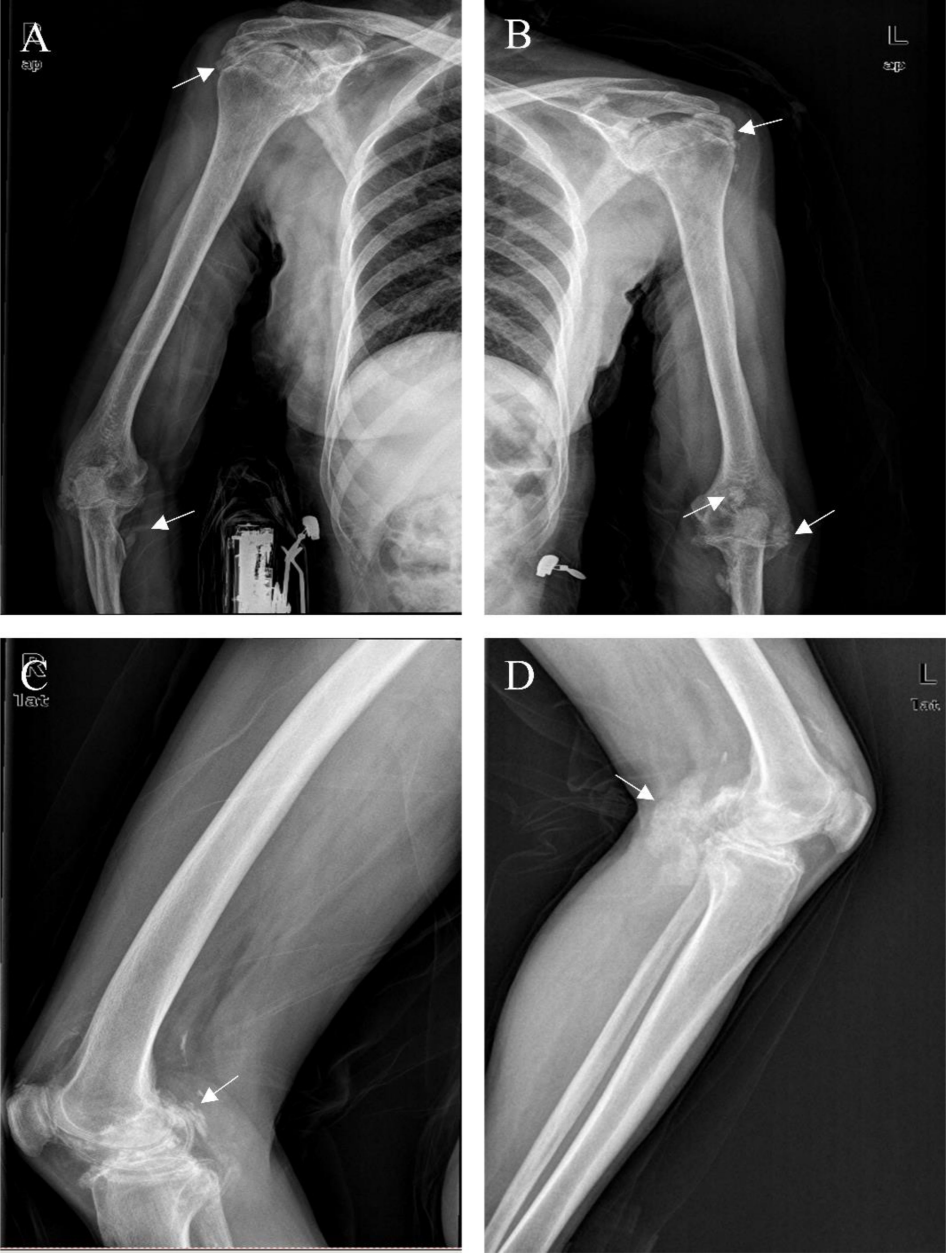
DR Images of the Limbs. Multiple instances of soft tissue calcifications in the patient were observed at and around the joints. We used the arrows point to some of these calcifications **(A-D)**.

Digital Radiography (DR): Multiple soft-tissue calcifications were evident in the extremity joints, particularly around the joints (**Figure 2**).

**Figure 3:**
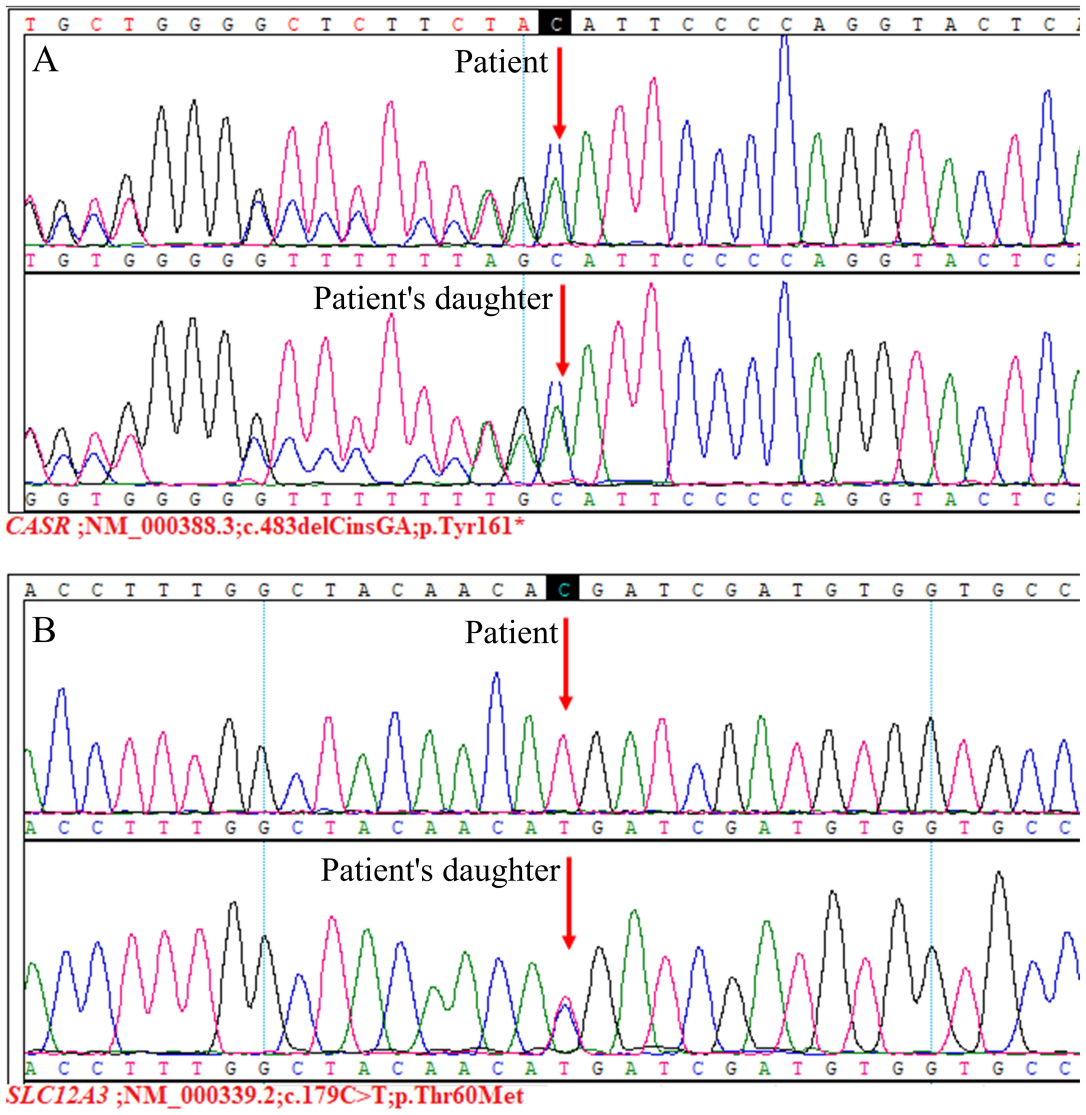
Genetic sequencing results. The *CASR*;NM_000388.3:c.483delCinsGA(p.Tyr161*) variant **(A)**, this variant was not reported previously. It was classified as pathogenic according to the ACMG guidelines (PVS1+PM2+PP4). Additionally, the *SLC12A3*;NM_000339.2:c.179C>T(p.Thr60Met) variant **(B)**, for which pathogenicity has been previously reported (7, 8). This variant was judged to be pathogenic based on ACMG guidelines (PS3+PM1+PM2+PM3_Strong+PP3+PP4).

In our patient, there was no evidence of primary hyperparathyroidism or malignancy. The presence of moderate hypercalcemia, normal or mildly elevated serum PTH levels, hypocalciuria, and hypophosphatemia suggested the possibility of FHH. Additionally, the CCCR were 0.004 and 0.003, further supporting the diagnosis of FHH. However, FHH could not account for the patient’s hypokalemia and hypomagnesemia. In this case, the patient exhibited hypokalemia, hypomagnesemia, and hypocalciuria, without hypochloremic metabolic alkalosis or hyperreninemic hyperaldosteronism. Our examination of the pertinent literature revealed that two primary diseases are characterized by hypocalciuria: FHH and GS (6). We also conducted a detailed investigation into the patient’s family and discovered that the patient’s daughter suffered from hypercalcemia, hypokalemia, hypomagnesemia, and hypocalciuria. Additionally, two of the patient’s three sisters were affected: one with hypercalcemia and the other with hypokalemia and hypomagnesemia. Considering the patient’s clinical presentation, laboratory findings, and family survey, we suspected the presence of comorbid FHH and GS. The patient and her daughter consented to genetic testing, which, through high-throughput sequencing and Sanger sequencing, detected heterozygous mutations in *CASR* and homozygous mutations in *SLC12A3* in the patient, and heterozygous mutations in both *CASR* and *SLC12A3* in the patient’s daughter (**Figure 3**).

**Figure 4:**
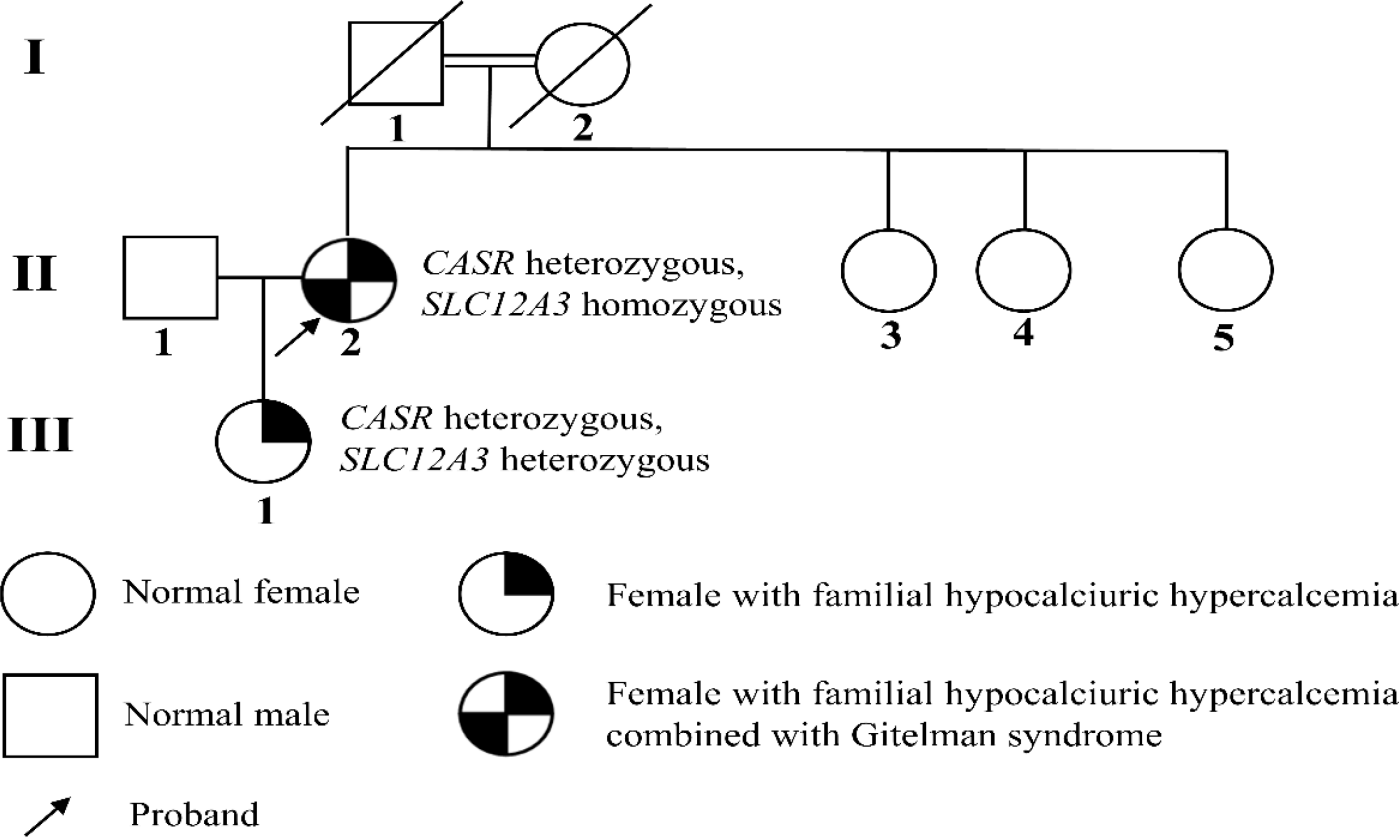
Pedigree of the family. GS is an autosomal recessive disorder caused by homozygous or compound heterozygous mutations. The patient’s daughter does not meet the diagnostic criteria for GS, and current evidence suggests she only carries the *SLC12A3* heterozygous disease-causing mutation, p.Thr60Met.

Based on the gene sequencing results and laboratory findings in the patient’s family, a family pedigree was constructed (**Figure 4**).

Combined with gene sequencing results, the patient was ultimately diagnosed with FHH1 and GS. The patient declined cinacalcet treatment due to financial constraints, exhibited intolerance to magnesium, which led to abdominal pain and diarrhea. The current medication regimen includes potassium chloride 3.0g, potassium magnesium aspartate 1.8g, and spironolactone 60mg daily. Additionally, the patient is advised to increase their intake of magnesium-rich foods. Long-term follow-up at our outpatient clinic showed that blood calcium levels stabilized at approximately 3.0 mmol/l, while potassium and magnesium levels were around 3.5 mmol/l and 0.35 mmol/l, respectively.

## Discussion

FHH1 is typically considered a rare disease, with its genetic prevalence estimated at 1.3 per 100,000. However, according to the DiscovEHR Cohort, the genetic prevalence of FHH1, including patients with normal blood calcium levels, is significantly higher at 74 per 100,000 (9). FHH1, which accounts for approximately 65% of all FHH cases, arises from germline heterozygous loss-of-function mutations in the CaSR gene on chromosome 3q21.1. The majority of these mutations are missense substitutions (10). The CaSR regulates calcium homeostasis mainly through its effects in the parathyroid gland and kidney. Activation by elevated circulating Ca^2+^ results in decreased secretion of PTH and reduced renal tubular Ca^2+^ reabsorption, respectively (11).

**Figure 5:**
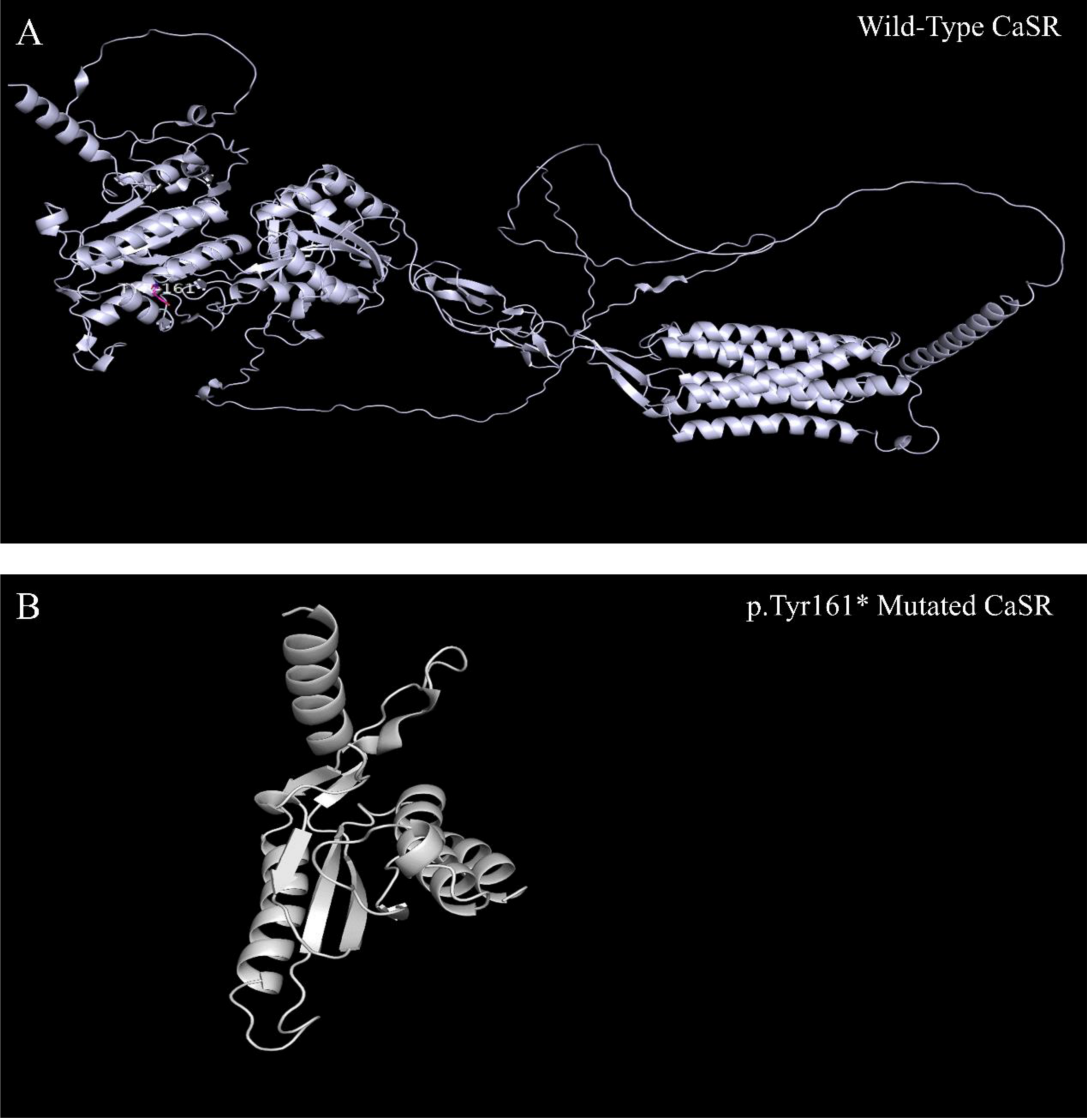
CaSR three-dimensional structure. As shown, compared to the wild-type CaSR protein **(A)**, the p.Tyr161* mutated CaSR protein is significantly truncated **(B)**.

This patient carries a heterozygous mutation, c.483delCinsGA(p.Tyr161*), in the *CASR* gene, resulting in the deletion of base C and the simultaneous insertion of GA at position 483 within the coding sequence. This alteration results in a change from tyrosine to a stop codon at amino acid 161, predicting a truncated CaSR protein. The three-dimensional (3D) structure of the CaSR protein was retrieved from the Protein Data Bank (PDB), and AF_AFP35670F1 was selected. The 3D structure of the wild-type CaSR protein and modifications to the mutant protein’s structure were both performed using PyMOL. It is evident that this mutation alters the protein structure and may lead to functional abnormalities (**Figure 5**).

If heterozygous missense or nonsense mutations inhibit receptor expression at the cell surface, approximately 50% of the receptors, provided by the wild-type allele, will continue to mediate parathyroid and renal calcium sensing. However, heterozygous mutations that allow CaSR expression but impair its function could lead to severe FHH by acting as dominant negatives of the wild-type CaSR. This is because only 25% of the wild-type homodimers are available to regulate parathyroid and renal function (12). In this case, the patient exhibited typical symptoms of hypercalcemia, along with mild clinical manifestations. We hypothesize that 50% of the normal receptors remained functional, mediating parathyroid and renal calcium sensing. Furthermore, consistent with the predicted structure alteration of p.Tyr161*, mutations such as R648X that leads to truncated proteins could result in reduced calcium responsiveness and hypercalcemia (13, 14).

GS is one of the most common hereditary disorders affecting potassium homeostasis, with its prevalence in Asia rising to an astonishing 10.3 per 10,000 people, and mutation prevalence reaching as high as 3% (15). The molecular mechanism of GS is defined by loss of function of NCC, encoded by *SLC12A3*. This loss interferes with the normal reabsorption of sodium and chloride in the DCT (16). This patient had the Thr60Met mutation in NCC, which inhibits Ste20-related proline- and alanine-rich kinase (SPAK)/oxidative stress responsive kinase-1 (OSR1)–mediated phosphorylation of NCC and alters NCC transporter activity *in vitro* (8). Thr60Met is one of the most common missense mutations in the *SLC12A3* gene and is likely the most prevalent mutation among Chinese patients with GS (7, 17).

CC, which has been a prominent clinical feature in this patient for many years, is noteworthy in this case. The relationship between FHH and CC remains unclear. Since Heath (18) first described the association between FHH and CC, only a few cases reported (6, 19). However, the prevalence of CC is known to increase with age (6). In contrast, the incidence of CC may be high in GS. Chotard et al. systematically screened 57 patients with GS for cartilage calcium deposits and discovered that chondrocalcinosis was observed in 79% of these patients (20). CC associated with GS has been linked to hypomagnesemia. Hypomagnesemia is believed to increase extracellular ionic inorganic pyrophosphate, a precursor to calcium pyrophosphate crystals. The deposition of these crystals in the articular cartilage leads to CC. Furthermore, magnesium supplementation may help reduce both chondrocalcinosis and the flares of joint pain (21). The current patient experienced joint stiffness at the age of 44 years. In the study by Chotard et al., the mean age at diagnosis of CC was 48 years, while the patients reported by Volpe and Alix et al. presented with CC-related symptoms at ages 58 and 50 years, respectively (6, 19, 20),. This suggests that FHH may work in conjunction with GS to accelerate the progression of the condition. Although the relationship between FHH and CC remains unclear, relevant case reports exist, and a correlation between FHH and CC in this patient cannot be excluded.

The patient described in this case had been living with diabetes mellitus for 18 years. According to relevant research, patients with FHH may not exhibit altered glucose metabolism (22). However, patients with GS may be predisposed to diabetes mellitus, which is associated with hypokalemia and hypomagnesemia (23, 24). Therefore, we suspected that the diabetes mellitus in our patient was related to GS.

FHH is generally considered a benign condition, and most patients do not require pharmacological or surgical intervention. However, cinacalcet may be administered on a case-by-case basis. Although it has been successfully used in cases of severe hypercalcemia, its utility in mild FHH is limited due to the potential for side effects, such as hypocalcemia, nausea, and vomiting, as well as the associated cost burden (25, 26). GS is also considered a benign disorder, there is no definitive treatment for GS; instead, supportive care is paramount. The primary treatment involves water and salt supplements, with potassium chloride as the salt supplement of choice. Additionally, magnesium supplementation is essential for treating hypomagnesemia and preventing CC (5).

This patient has both FHH and GS, providing a unique opportunity to explore the potential mechanisms underlying the interaction between these two rare disorders. CaSR is expressed in various tissues but is predominantly found in the parathyroid glands and kidneys. It is expressed throughout the kidneys and plays a role in several segments, including the regulation of renin release, phosphate excretion, and the transport of ions such as Na^+^, Cl^-^, Ca²^+^, and Mg²^+^ (10, 27, 28). Loss-of-function mutations in the *CASR* gene in the parathyroid gland increase the set point for calcium sensing, necessitating higher-than-normal serum calcium levels to reduce PTH release. In FHH, renal sensing of serum calcium and downstream effects beyond the CaSR are also abnormal, clinically leading to hypocalciuria or potentially abnormal urinary calcium levels. Furthermore, the location and role of CaSR in the kidney have not been fully elucidated (27). The NCC is expressed in the renal DCT and plays a key role in Na^+^, Cl^-^, and K^+^ homeostasis, as well as in blood pressure regulation (29). Loss of NCC function due to mutations in the *SLC12A3* gene disrupts NaCl reabsorption in the DCT, leading to a reduction in vascular volume and subsequent increases in renin and aldosterone secretion. Elevated aldosterone levels enhance electrogenic sodium reabsorption via effects on epithelial sodium channel (ENaC) in the collecting ducts, while also increasing the excretion of potassium and hydrogen ions, resulting in hypokalemia and metabolic alkalosis (30). Hypocalciuria in GS results from increased Ca²^+^ reabsorption to compensate for salt loss, while the cause of hypomagnesemia remains unknown. One of the most widely accepted hypotheses is an alteration in the expression of the transient receptor potential cation channel subfamily m member 6 (TRPM6) (5). Both CaSR and NCC are expressed in the kidney, with overlapping roles in ion transport. Theoretically, FHH and GS may influence each other’s phenotypes, but since CaSR is expressed in various tissues and throughout the kidney, its influence may be broader, potentially having a greater impact on the phenotype of GS. The absence of hypochloremic metabolic alkalosis and hyperreninemic aldosteronism suggests that the *CASR* mutation in this patient may have altered the renal tubular response to the electrolyte imbalances characteristic of GS. However, further functional studies are needed to determine whether this mutation exacerbates or modulates the GS phenotype, potentially revealing novel pathways for ion transport regulation.

In addition, the discovery of novel mutations in the *CASR* gene has important implications for genetic counseling. The fact that the parents of our patient were cousins underscores the need for a detailed family history and genetic testing of offspring from consanguineous marriages, as this may lead to the identification of previously unreported diseases. From a management perspective, the simultaneous occurrence of FHH and GS presents distinct challenges. The characteristic hypokalemia and hypomagnesemia of GS were more detrimental to our patient than FHH, and both ionic disturbances may predispose the patient to diabetes mellitus and hypomagnesemia is associated with CC, which can be both prevented and alleviated by magnesium supplementation. In clinical practice, prioritizing the correction of hypokalemia and hypomagnesemia is advisable when treating patients with a combination of FHH and GS. In particular, regular monitoring of ion levels is essential and enables the development of individualized treatment plans tailored to the patient’s genotypic and phenotypic features.

## Conclusion

A case of FHH1 associated with GS is reported for the first time. This case report suggests that multiple genetic disorders can coexist in patients with complex clinical phenotypes. The heterozygous dominant mutation in the *CASR* gene and the homozygous recessive mutation in the *SLC12A3* gene in this patient represent the molecular genetic basis for the co-occurrence of FHH and GS. This provides new evidence and ideas into the molecular mechanisms underlying the co-occurrence of multiple metabolic genetic disorders and clinical diagnosis and treatment. Protein molecular modeling demonstrated that the novel mutations in the *CASR* gene cause structural alterations in the CaSR protein, thereby impairing its function. In addition, the novel *CASR* mutation highlights the importance of genetic counseling for the offspring of consanguineous marriages. There may be an association between CC and FHH in this patient; however, further investigation is needed to confirm this, as well as to elucidate the mechanism by which CC occurs in FHH. This case also explores the potential mechanistic link between FHH and GS, as well as the role CaSR may play in broader electrolyte regulation. Clinically, the significant risks associated with GS-related hypokalemia and hypomagnesemia in patients with both FHH and GS necessitate a proactive, patient-centered approach. Finally, genetic testing and molecular diagnostics are especially important in the pediatric and adolescent populations, given the potential for complications in adulthood. Further studies involving multiple pedigrees, multicenter research, and molecular mechanisms will help elucidate the genotypic and phenotypic relationships between FHH and GS comorbidities.

## Data Availability

The original contributions presented in the study are included in the article/**Supplementary Material**. Further inquiries can be directed to the corresponding author.
