# Supplementary material for: Case Report: Familial hypocalciuric hypercalcemia type 1 with a novel mutation combined with Gitelman syndrome and a review of the literature
Source: Front Endocrinol (Lausanne). 2025 Feb 25;16:1503128. doi: 10.3389/fendo.2025.1503128 (PMC11893564; doi:10.3389/fendo.2025.1503128)
Supplement: Supplementary file 1 [file DataSheet1.docx]

**Supplementary Table 1** Additional laboratory test results.

| Variables | Value | Reference Range |
| --- | --- | --- |
| Alpha-fetoprotein (ng/ml) | 2.76 | 0.00–7.00 |
| Carcinoembryonic Antigen (ng/ml) | 0.69 | 0.00–3.40 |
| Carbohydrate Antigen 19-9 (U/ml) | 13.64 | 0.00–39.00 |
| Carbohydrate Antigen 12-5 (U/ml) | 6.8 | 0.00–35.00 |
| Carbohydrate Antigen 15-3 (U/ml) | 3.17 | 0.00–25.00 |
| Free Triiodothyronine (pmol/L) | 5.18 | 3.50–6.50 |
| Free Thyroxine (pmol/L) | 16.93 | 11.50–22.70 |
| Thyroid-Stimulating Hormone (mIU/L) | 2.09 | 0.51–4.94 |
| Anti-Thyroglobulin Antibody (U/ml) | 71.82 | 0.00–60.00 |
| Anti-Thyroid Peroxidase Antibody (U/ml) | <28 | 0.00–60.00 |
| Growth Hormone (ng/ml) | <0.05 | <10.00 |
| Cortisol (μg/dl) | 19.83 | 4.30–22.40 |
| Luteinizing Hormone (mIU/L) | 13.01 | Follicular phase: 2.4–12.6; Ovulatory phase: 14–95; Luteal phase: 1.0–11.4; Postmenopausal: 7.7–58.5 |
| Follicle-Stimulating Hormone (mIU/L) | 33.35 | Follicular phase: 3.5–12.5; Ovulatory phase: 4.7–21.5; Luteal phase: 1.7–7.7; Postmenopausal: 25.8–134.8 |
| Testosterone (nmol/L) | 0.11 | 0.22–2.90 |
| Progesterone (nmol/L) | 0.97 | Follicular phase: 0.6–4.7; Ovulatory phase: 2.4–9.4; Luteal phase: 5.3–86; Postmenopausal: 0.3–2.5 |
| Estradiol (pmol/L) | 25.29 | Follicular phase: 90.7–716; Ovulatory phase: 243–1509; Luteal phase: 147–960; Postmenopausal: 36.7–145 |
| Prolactin (ng/ml) | 16.81 | 3.4–24.1 |
| Serum Kappa Light Chain (mg/dl) | 1267 | 629–1350 |
| Serum Lambda Light Chain (mg/dl) | 645 | 313–723 |
| Aldosterone (pg/ml) | 130 | 10–160 |
| Renin (pg/ml) | 19 | 2–24 |
| Angiotensin II (pg/ml) | 40 | 28–52 |
| Alkaline Phosphatase (U/L) | 48 | 50–135 |
| Urea (mmol/L) | 2.71 | 1.70–7.14 |
| Creatinine (μmol/L) | 34 | 44–115 |
| Arterial Blood pH | 7.404 | 7.35–7.45 |
| Arterial Partial Pressure of CO₂ (mmHg) | 44 | 35–45 |
| Arterial Partial Pressure of O₂ (mmHg) | 77.8 | 80–100 |
| Actual Bicarbonate (mmol/L) | 26.9 | 21.0–28.0 |
| Standard Bicarbonate (mmol/L) | 26.0 | 21.0–25.0 |
| Base Excess (mmol/L) | 1.8 | -3.0–3.0 |
| Urine pH | 8.0 | 5.4–8.4 |
| Urine Specific Gravity | 1.010 | 1.003–1.030 |
| Serum Osmolality (mOsm/kg) | 288 | 280–310 |
| Urine Osmolality (mOsm/kg) | 849 | 700–1000 |
| Urinary Microalbumin/Creatinine Ratio (mg/g) | 12.4 | <30 |

The laboratory data in this table were all measured in 2014, and the normal range refers to the standard values established by the laboratory of our hospital.
