# Supplementary material for: Case Report: Familial hypocalciuric hypercalcemia type 1 with a novel mutation combined with Gitelman syndrome and a review of the literature
Source: Front Endocrinol (Lausanne). 2025 Feb 25;16:1503128. doi: 10.3389/fendo.2025.1503128 (PMC11893564; doi:10.3389/fendo.2025.1503128)
Supplement: Supplementary file 2 [file SupplementaryFile1.pdf]

[redacted] 姓名[redacted] 住院号码:[redacted] 样本种类: 静脉血 样本编号: 33
性别: 女 科 室: 内分泌科病房 采样日期: 2014.05.26 0[illegible] 样本状态: 正常
年龄: 60 岁 病人床号:[redacted] 送检医生:[redacted] 备注信息:

No	项 目	结 果	参考区间	单位
1	餐后2小时胰岛素	216.0		mU/L
2	餐后2小时C肽	10.37		ng/ml

此线以下无检验项目

日期: 14.05.26 09:54 报告日期: 14.05.26 10:49 检验医生:[redacted] 审核医生:[redacted]

此结果仅对所检测样本负责，样本保留72小时，如有疑问请及时与检验科联系。

2[redacted] [illegible]

姓名[redacted] 住院号码：[redacted] 条码号：1403542969

性别：女 科 室：内分泌科病房 样本种类：静脉血 样本编号：508

年龄：60 岁 床 号：[redacted] 采样日期：14.05.26 08 样本状态：正常

送检医生：[redacted] 备注信息：

No	项 目	结 果		参考区间	单位
1	餐后2小时血糖	15.37	↑	4.40-7.80	mmol/L

此线以下无检验项目

No	项 目	结 果	参考区间	单位

检验日期：14.05.26 09:40 报告日期：14.05.26 10:27 检验者：[redacted] 审核者：[redacted]

此结果仅对所检测样本负责，样本保留72小时，如有疑问请及时与检验科联系。

大连医科大学附属第一医院核医学科

ECT 报告

检查日期： 2014 年 05 月 27 日　　　　**检 查 号：** [redacted]

**姓　　名：**	[redacted]	**性　　别：**	女	**年　　龄：**	59 岁
**住 院 号：**	-	**科　　别：**	普通外科门诊		
**临床诊断：**	甲状旁腺良性肿瘤	**检查项目：**	甲状旁腺显像		
**显 像 剂：**	99Tc-MIBI	**剂　　量：**	370MBq	**采集方式：**	平面

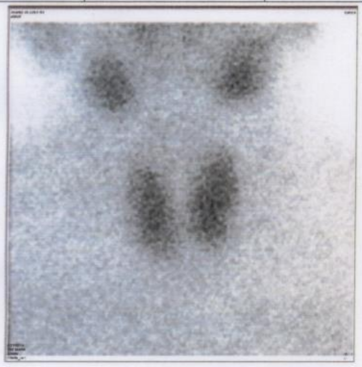

20min

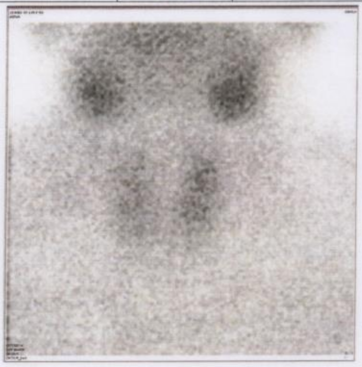

2h

检查方法及所见：

静脉注射显像剂，20 分钟、2 小时后，分别进行颈部前位初始相及延迟相采集。

初始相：可见双侧甲状腺显影，其位置、形态正常，放射性分布欠均匀；颈部其余部位及上胸部未见异常放射性分布。

延迟相：双侧甲状腺放射性分布正常“洗脱”，颈部其余部位及上胸部未见异常放射性分布。

诊断意见：

甲状旁腺 MIBI 显像未见异常。

报告医师： [redacted]　　**审核医师：** [redacted]　　**报告日期：** 2014 年 05 月 27 日

联系电话：83635963-3246

OTOsuite

[redacted]
年龄:
出生日期:
报告日期: 2014/5/19
测试者:

大连市中心医院

声阻抗报告单

注：该检测结果仅反映受试者检测当时之情况。

右 (2014/5/19)

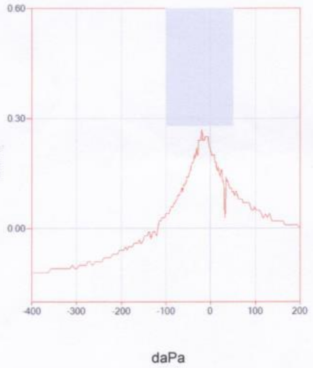

左 (2014/5/19)

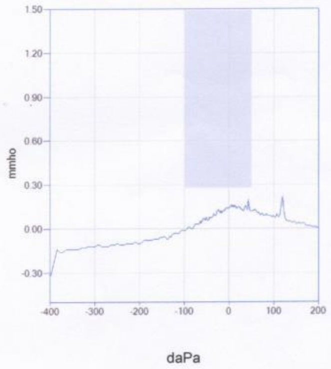

鼓室图		
纯音	226	Hz
SA	0.25	mmho
TPP	-13	daPa
ECV	0.71	ml
TW	83	daPa
类型	AS	

鼓室图		
纯音	226	Hz
SA	0.14	mmho
TPP	21	daPa
ECV	0.84	ml
TW	138	daPa
类型	AS	

反射	阈值(dB HL)					衰减(秒)	
	500	1k	2k	4k	宽频带噪音	500	1k
右耳同侧							
左耳同侧							
右耳对侧							

[redacted] [大连市中心医院]检验报告单【免疫】

条码号：1403578898

病人姓名：[redacted]	住院号码：[redacted]	样本种类：卧位	样本编号：608
病人性别：女	病人科室：[redacted]	采样日期：2014.06.04 04	样本状态：正常
病人年龄：60 岁	病人床号：[redacted]	送检医生：[redacted]	备注信息：

No	项 目	结 果	参考区间	单位
1	醛固酮	130	10-160	pg/ml
2	血管紧张素II	40	28-52	pg/ml

此线以下无检验项目

[illegible]日期：14.06.04 09:06　报告日期：14.06.04 11:10　检验医生：张萱　审核医生：兰孝芹

此结果仅对所检测样本负责，样本保留72小时，如有疑问请及时与检验科联系。

条码号：1403504050

姓名：[redacted]	住院号码：[redacted]	样本种类：静脉血	样本编号：145
性别：女	科　室：[redacted]	采样日期：14.05.14 05	样本状态：已复查
年龄：60　岁	床　号：0757	送检医生：[redacted]	备注信息：

No	项　目	结　果		参考区间	单位
	丙氨酸氨基转移酶	27		7-40	U/L
	天冬氨酸氨基转移酶	23		13-35	U/L
	总蛋白	71.3		65.0-85.0	g/L
	白蛋白	42.1		40.0-55.0	g/L
	L-r-谷氨酰基转移酶	15		7-45	U/L
	碱性磷酸酶	48	↓	50-135	U/L
	总胆红素	14.4		5.1-19.0	umol/L
	直接胆红素	4.3		1.7-6.8	umol/L
	总胆汁酸	39	↑	0-10	umol/L
	前白蛋白	241		180-390	mg/L
	葡萄糖	6.32	↑	3.80-6.10	mmol/L
	尿素	2.71		1.70-7.14	mmol/L
	肌酐	34	↓	44-115	umol/L
	尿酸	239		150-360	umol/L
	胱抑素C	0.77		0.00-1.03	mg/L
	总胆固醇	3.78		3.11-5.18	mmol/L

此线以下无检验项目

No	项　目	结　果		参考区间	单位
17	三酰甘油	0.78		0.56-1.70	mmol/L
18	高密度脂蛋白胆固醇	1.19		1.04-1.55	mmol/L
19	低密度脂蛋白胆固醇	1.88		健康人群 2.07-3.12 高危人群 <2.58 极高危人群 <2.07	mmol/L
20	钾	2.73	↓↓	3.50-5.30	mmol/L
21	钠	137.7		137.0-147.	mmol/L
22	氯化物	98.3	↓	99.0-110.C	mmol/L
23	碳酸氢根	26.7		22.0-29.0	mmol/L
24	总钙	3.08	↑	2.10-2.60	mmol/L
25	无机磷	0.75	↓	0.80-1.50	mmol/L
26	镁	0.52	↓	0.66-1.07	mmol/L

此线以下无检验项目

[illegible]日期：14.05.14 08:02　报告日期：14.05.14 09:38　检验者：[redacted]　审核者：[redacted]

此结果仅对所检测样本负责，样本保留72小时，如有疑问请及时与检验科联系。

[illegible]

姓名：[redacted]	住院号码：[redacted]	样本种类： 静脉血	样本编号： 262
性别： 女	科　室： 神经内科一病房	采样日期： 14.05.16 05	样本状态： 正常
年龄： 60　岁	床　号：[redacted]	送检医生：[redacted]	备注信息：

No	项　目	结　果		参考区间	单位
1	钾	3.11	↓	3.50-5.30	mmol/L
2	钠	135.6	↓	137.0-147.	mmol/L
3	氯化物	100.0		99.0-110.C	mmol/L

此线以下无检验项目

[illegible]验日期：14.05.16 08:50　报告日期：14.05.16 10:05　检验者：[redacted]　审核者：[redacted]

此结果仅对所检测样本负责，样本保留72小时，如有疑问请及时与检验科联系。

姓名: [redacted]	住院号码: [redacted]		样本种类: 静脉血		样本编号: 176
性别: 女	科　室: 神经内科一病房		采样日期: 14.05.20 05		样本状态: 正常
年龄: 60　岁	床　号: [redacted]		送检医生: [redacted]		备注信息:

No	项目	结果		参考区间	单位
1	钾	3.47	↓	3.50-5.30	mmol/L
2	钠	140.6		137.0-147.	mmol/L
3	氯化物	102.1		99.0-110.(	mmol/L
4	总钙	3.07	↑	2.10-2.60	mmol/L
5	无机磷	0.85		0.80-1.50	mmol/L
6	镁	0.49	↓	0.66-1.07	mmol/L

此线以下无检验项目

No	项目	结果	参考区间	单位

检验日期: 14.05.20 07:58　报告日期: 14.05.20 09:27　检验者: [redacted]　审核者: [redacted]

此结果仅对所检测样本负责，样本保留72小时，如有疑问请及时与检验科联系。

姓名：[redacted]　住院号码：[redacted]　样本种类：尿　样本编号：465
性别：女　科　室：神经内科一病房　采样日期：14.05.21 05　样本状态：正常
年龄：60　岁　床　号：[redacted]　送检医生：[redacted]　备注信息：

No	项　目	结　果		参考区间	单位
1	24小时尿总钙定量	2.13	↓	2.50-7.50	mmol/24
2	24小时尿无机磷定量	15.1	↓	22.0-48.0	mmol/24

此线以下无检验项目

No	项　目	结　果	参考区间	单位

检验日期：14.05.21 09:22　报告日期：14.05.21 12:01　检验者：[redacted]　审核者：[redacted]

此结果仅对所检测样本负责，样本保留72小时，如有疑问请及时与检验科联系。

姓名：[redacted] 住院号码：[redacted] 样本种类：静脉血 样本编号：217

性别：女 科 室：内分泌科病房 采样日期：14.05.26 04 样本状态：已复查

年龄：60 岁 床 号：[redacted] 送检医生：[redacted] 备注信息：

No 项 目	结 果		参考区间	单位
碱性磷酸酶	47	↓	50-135	U/L
总胆汁酸	8		0-10	umol/L
葡萄糖	5.72		3.80-6.10	mmol/L
钾	2.80	↓↓	3.50-5.30	mmol/L
钠	139.2		137.0-147.	mmol/L
氯化物	97.6	↓	99.0-110.C	mmol/L
碳酸氢根	30.9	↑	22.0-29.0	mmol/L
总钙	3.04	↑	2.10-2.60	mmol/L
无机磷	0.84		0.80-1.50	mmol/L
镁	0.39	↓	0.66-1.07	mmol/L
肌酸激酶同工酶-MB	19		0-24	U/L
肌酸激酶	43		0-173	U/L
糖化血清白蛋白%	13.3		11.0-16.0	%

此线以下无检验项目

No 项 目	结 果	参考区间	单位

日期：14.05.26 08:27 报告日期：14.05.26 09:40 检验者：[redacted] 审核者：[redacted]

此结果仅对所检测样本负责，样本保留72小时，如有疑问请及时与检验科联系。

姓名: [redacted]	住院号码: [redacted]	样本种类: 尿	样本编号: 483
性别: 女	科　室: 内分泌科病房	采样日期: 14.05.26 06	样本状态: 正常
年龄: 60 岁	床　号: 0546	送检医生: [redacted]	备注信息:

No	项　目	结　果		参考区间	单位
	24小时尿肌酐定量	3.52	↓	7.00-18.0C	mmol/24l
	24小时尿钾定量	27.8		25.0-100.C	mmol/24l
	24小时尿钠定量	127.5	↓	130.0-260.	mmol/24l
	24小时尿氯化物定量	113.8	↓	170.0-255.	mmol/24l
	24小时尿总钙定量	1.37	↓	2.50-7.50	mmol/24l
	24小时尿无机磷定量	9.3	↓	22.0-48.0	mmol/24l

此线以下无检验项目

Cr/Ca ~~0.385~~ 0.138

日期: 14.05.26 09:39　报告日期: 14.05.26 10:35　检验者: [redacted]　审核者: [redacted]

此结果仅对所检测样本负责，样本保留72小时，如有疑问请及时与检验科联系。

第　　页

[redacted] 住院号码：[redacted] 样本种类：静脉血 条码号：1403563228

别：女 科 室：内分泌科病房 采样日期：14.05.29 04 样本编号：340

龄：60 岁 床 号：[redacted] 送检医生：[redacted] 样本状态：正常

备注信息：

o 项 目	结 果		参考区间	单位
碱性磷酸酶	51		50-135	U/L
钾	3.03	↓	3.50-5.30	mmol/L
钠	139.8		137.0-147.	mmol/L
氯化物	98.8	↓	99.0-110.(	mmol/L
碳酸氢根	28.8		22.0-29.0	mmol/L
总钙	3.01	↑	2.10-2.60	mmol/L
无机磷	0.79	↓	0.80-1.50	mmol/L
镁	0.39	↓	0.66-1.07	mmol/L

此线以下无检验项目

No 项 目	结 果	参考区间	单位

日期：14.05.29 08:54 报告日期：14.05.29 09:43 检验者：[redacted] 审核者：[redacted]

JY15

条码号：1403570943

名：[redacted] 住院号码：[redacted] 样本种类：静脉血 样本编号：270

别：女 科 室：内分泌科病房 采样日期：14.06.03 05 样本状态：正常

龄：60 岁 床 号：[redacted] 送检医生：[redacted] 备注信息：

No	项 目	结 果		参考区间	单位
	丙氨酸氨基转移酶	14		7-40	U/L
	天冬氨酸氨基转移酶	18		13-35	U/L
	L-r-谷氨酰基转移酶	12		7-45	U/L
	碱性磷酸酶	45	↓	50-135	U/L
	葡萄糖	5.55		3.80-6.10	mmol/L
	尿素	4.28		1.70-7.14	mmol/L
	肌酐	33	↓	44-115	umol/L
	尿酸	263		150-360	umol/L
	总胆固醇	3.31		3.11-5.18	mmol/L
	三酰甘油	0.77		0.56-1.70	mmol/L
	高密度脂蛋白胆固醇	1.03	↓	1.04-1.55	mmol/L
	低密度脂蛋白胆固醇	1.73		健康人群 2.07-3.12 高危人群 <2.58 极高危人群 <2.07	mmol/L
	钾	2.98	↓	3.50-5.30	mmol/L
	钠	139.2		137.0-147.	mmol/L
	氯化物	102.9		99.0-110.C	mmol/L
	碳酸氢根	30.2	↑	22.0-29.0	mmol/L
17	总钙	2.87	↑	2.10-2.60	mmol/L
18	无机磷	0.88		0.80-1.50	mmol/L
19	镁	0.45	↓	0.66-1.07	mmol/L
20	肌酸激酶	49		0-173	U/L

此线以下无检验项目

日期：14.06.03 08:58 报告日期：14.06.03 09:39 检验者：[redacted] 审核者：[redacted]

此结果仅对所检测样本负责，样本保留72小时，如有疑问请及时与检验科联系。

[illegible]名：[redacted]	住院号码：[redacted]	样本种类：尿	样本编号：345
[illegible]别：女	科　室：内分泌科病房	采样日期：14.06.03 07	样本状态：正常
[illegible]龄：60　岁	床　号：[redacted]	送检医生：[redacted]	备注信息：

No	项　目	结　果		参考区间	单位
	24小时尿肌酐定量	7.48		7.00-18.0C	mmol/24h
	24小时尿尿酸定量	3.09		2.40-5.90	mmol/24h
	24小时尿钾定量	77.8		25.0-100.C	mmol/24h
	24小时尿钠定量	194.4		130.0-260.	mmol/24h
	24小时尿氯化物定量	210.6		170.0-255.	mmol/24h
	24小时尿总钙定量	1.66	↓	2.50-7.50	mmol/24h
	24小时尿无机磷定量	20.2	↓	22.0-48.0	mmol/24h
	24小时尿葡萄糖定量	0.04	↓	0.56-5.00	mmol/24h

此线以下无检验项目

[illegible]日期：14.06.03 09:04　报告日期：14.06.03 10:55　检验者：[redacted]　审核者：[redacted]

此结果仅对所检测样本负责，样本保留72小时，如有疑问请及时与检验科联系。

条码号：[illegible]

姓名：[redacted] 住院号码：[redacted] 样本种类： 静脉血 样本编号： 768

性别： 女 科 室： 内分泌科病房 采样日期： 14.06.05 05 样本状态： 正常

年龄： 60 岁 床 号：[redacted] 送检医生：[redacted] 备注信息：

No	项 目	结 果		参考区间	单位
1	钾	3.52		3.50-5.30	mmol/L
2	钠	140.1		137.0-147.	mmol/L
3	氯化物	103.1		99.0-110.(	mmol/L
4	碳酸氢根	24.1		22.0-29.0	mmol/L
5	总钙	2.88	↑	2.10-2.60	mmol/L
6	无机磷	0.76	↓	0.80-1.50	mmol/L
7	镁	0.39	↓	0.66-1.07	mmol/L

此线以下无检验项目

No	项 目	结 果	参考区间	单位

姓名：[redacted] 住院号码：[redacted] 样本种类：动脉血 样本编号：7
性别：女 科 室：内分泌科病房 采样日期：14.05.26 08 样本状态：正常
年龄：60 岁 床 号：[redacted] 送检医生：[redacted] 备注信息：

No	项 目	结 果		参考区间	单位
	血液酸碱度	7.404		7.35-7.45	
	动脉二氧化碳分压	44.0		35-45	mmHg
	动脉血氧分压	77.8	↓	80-100	mmHg
	实际碳酸氢盐浓度	26.9		21.0-28.0	mmol/L
	标准碳酸氢盐浓度	26.0	↑	21.0-25.0	mmol/L
	缓冲碱	1.8		-3.0-3.0	mmol/L
	细胞外液剩余碱	2.2		-3.0-3.0	mmol/L
	二氧化碳总量	28.2	↑	23.0-28.0	mmol/L
	总血红蛋白	13.6		11.5-17.4	g/dL
	动脉血氧饱和度	95.5		90.0-98.0	%
	氧和血红蛋白	95.0		95.0-99.0	%
	碳氧血红蛋白	0.2	↓	0.5-2.5	%
	高铁血红蛋白	0.3	↓	0.4-1.5	%
	脱氧血红蛋白	4.5		1.0-5.0	%
	pAtm	730.0			mmHg

此线以下无检验项目

No	项 目	结 果	参考区间	单位

日期：14.05.26 07:55 报告日期：14.05.26 09:08 检验者[redacted] 审核者：[redacted]

此结果仅对所检测样本负责，样本保留72小时，如有疑问请及时与检验科联系。

辽宁省大连市中心医院

病人:	[redacted]			
生日:	1954-6-15 59.9 年			
身高 / 体重:	154.0 cm 42.0 kg	测量:	2014-5-29 15:14:59	(11.20)
性别 / 种族:	女性 亚裔人	分析:	2014-5-29 15:15:09	(11.20)

正位脊柱 骨密度

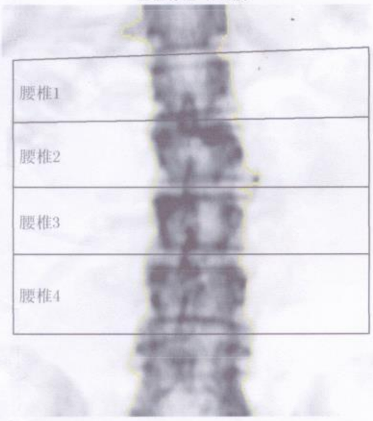

骨密度仪参考: L1-L4

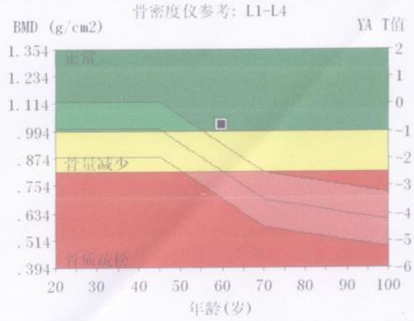

区域	骨密度[1] (g/cm2)	年轻成人[2] T-值评分	与同年龄正常人群 Z-值评分
腰椎1	.854	-1.5	.9
腰椎2	1.035	-.6	2.0
腰椎3	1.073	-.7	1.9
腰椎4	1.084	-.5	1.7
L1-L4	1.022	-.8	1.7

建议:

腰椎骨量减少

影像不用作诊断

打印: 2014-5-29 15:15:14 (11.20)76:3.00:50.03:12.0 0.00:8.04 0.60x1.05
16.2:%脂肪=21.9%
0.00:0.00 0.00:0.00
文件名: zqqb6n6g9g.dfs
扫描模式: 标准 37.0 μGy

1 -在统计上68% 的重复扫描将在1个标准差内。 (+/-0.010 g/cm2 for 正位脊柱 L1-L4)
2 -中国 (年龄20-40) 正位脊柱 参考人群 (v110)
3 -用年龄, 体重(女性 25-100 kg), 种族比较校正
11 -World Health zation世界卫生组织-定义白种女性骨质疏松及骨含量减少标准: 正常=T值>-1.0SD;骨含量减少=T值在-1.0--2.5SD之间; 骨质疏松=T值<-2.5SD;(WHO利用白种年轻妇女的正常值定义T值)

辽宁省大连市中心医院

病人:	[redacted]				
生日:	1954-6-15	59.9 年			
身高 / 体重:	154.0 cm	42.0 kg	测量:	2014-5-29 15:16:12	(11.20)
性别 / 种族:	女性	亚裔人	分析:	2014-5-29 15:16:13	(11.20)

右侧股骨 骨密度

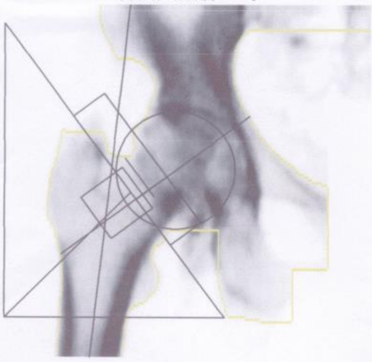

骨密度仪参考: 全部

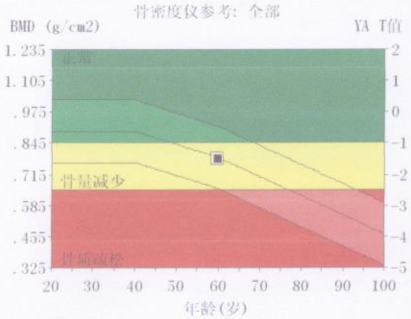

区域	骨密度[1] (g/cm2)	年轻成人[2] T-值评分	与同年龄正常人群 Z-值评分
颈	.870	-.5	1.4
Wards三角	.576	-2.0	.2
大粗隆	.592	-1.5	.0
全部	.778	-1.5	-.1

髋关节中轴长度比较(mm)

右侧= -5.2

-30 -20 -10 均值 10 20 30

(右侧= 92.6 mm) (均值 = 97.8 mm)

建议:

右侧股骨骨量减少

影像不用作诊断

打印: 2014-5-29 15:16:22 (11.20)76:0.75:50.03:12.0 0.00:9.60 0.60x1.05
13.8:%脂肪=17.4%
0.00:0.00 0.00:0.00
股骨颈角度 (度) = 53
核实在骨盆和股骨干之间有足够的分离。
文件名: itqb6n6g9g.dff
扫描模式: 薄 9.0 μGy

1 -在统计上68% 的重复扫描将在1个标准差内。 (+/-0.012 g/cm2 for 右侧股骨 全部)
2 -中国 (年龄20-40) 股骨 参考人群 (v110)
3 -用年龄, 体重(女性 25-100 kg), 种族比较校正
11 -World Health zation世界卫生组织-定义白种女性骨质疏松及骨含量减少标准: 正常=T值>-1.0SD;骨含量减少=T值在-1.0—2.5SD之间; 骨质疏松=T值<-2.5SD;(WHO利用白种年轻妇女的正常值定义T值)

GE Healthcare

Lunar Prodigy
DF+301012

大连市中心医院

彩色超声多普勒诊断报告单

ZXYY/CX-11-JL-28

住院号:[redacted]　　姓　名:[redacted]　　性　别:女　　年　龄:59　岁

检查部位:[乳腺及腋窝]　　申请科室:内分泌科病房

检查所见:

双侧乳腺腺体稍厚，层次结构紊乱，内呈网格状回声，分布不均，左侧乳腺局部导管扩张，内径约0.23cm，右侧乳腺局部导管扩张，内径约0.25cm。
左侧乳腺可探及实性结节，位于外象限3点，大小约0.55×0.45cm,距乳头约2.0cm,距体表约0.64cm,边界清晰,形态规整，后方回声不变。CDFI:内部及周边未见血流信号。
双侧腋窝探查，未见明显肿大淋巴结。

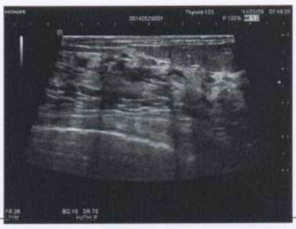

诊断:

双侧乳腺增生并局部导管扩张
左侧乳腺实性占位(BI-RADS 3级)

报告医师:[redacted]

报告时间:2014-05-29

医师签字:

大连市中心医院

彩色超声多普勒诊断报告单

ZXYY/CX-11-JL-28

住院号:[redacted] 姓 名:[redacted] 性 别:女 年 龄:59 岁

检查部位:[肝 胆 脾 胰 双肾] 申请科室:内分泌科病房

检查所见:

肝脏大小正常,右叶斜径约12.5cm, 表面光滑,肝缘锐利,实质回声均匀,血管走行正常,肝内外胆管未见扩张。
门静脉主干内径约1.0cm。
胆囊大小正常,壁厚约0.3cm,边缘光滑。
胆总管未见扩张, 内径约0.6cm。
脾脏大小正常,内部回声均匀。
胰腺大小、形态正常,回声均匀,主胰管未见扩张。
左肾大小约11.0x5.2cm,包膜完整,实质回声均匀,集合系统未见异常。
右肾大小约10.1x4.7cm,包膜完整,实质回声均匀,集合系统未见异常。

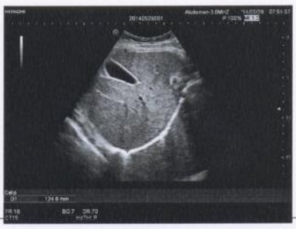

诊断:

肝胆脾胰双肾未见明显异常

报告医师:[redacted]

报告时间:2014-05-29

医师签字:

大连市中心医院

彩色超声多普勒诊断报告单

ZXYY/CX-11-JL-29

住院号:[redacted] 姓 名:[redacted] 性 别:女 年 龄:59 岁

检查部位:[子宫 附件] 申请科室:内分泌科病房

检查所见:

子宫全切除术后。
双侧卵巢未显示。
左侧附件区见大小约32x20x20mm囊肿，形态规则，边界清晰，内呈液性，其内及周边未见明显血流信号。
盆腔未见明显积液影像。

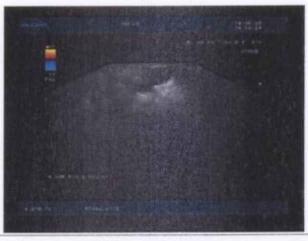

诊断: 左侧附件区囊肿

报告医师:[redacted]

报告时间:2014-05-26

医师签字:

姓名：[redacted] 住院号码：[redacted] 样本种类：上午 样本编号：9
性别：女 科 室：内分泌科病房 采样日期：2014.05.26 0 样本状态：正常
年龄：60 岁 病人床号：[redacted] 送检医生：[redacted] 备注信息：

No	项 目	结 果	参考区间	单位
1	皮质醇	19.83	4.30-22.40	ug/dl

此线以下无检验项目

日期：14.05.26 09:52 报告日期：14.05.26 10:30 检验医生：[redacted] 审核医生：[redacted]

此结果仅对所检测样本负责，样本保留72小时，如有疑问请及时与检验科联系。

大连市中心医院放射科
CT诊断报告单

ZXYY/CX-11-JL-27　　　　辽宁省医学影像检查结果互认单位

患者类型：住院　　患者编号：[redacted]　　检查号：[redacted]

姓名：[redacted]　　性别：女　　年龄：59

科　别：内分泌科病房　　设备：GE CT64

检查部位：［双肺,平扫］

胸部MSCT平扫

检查所见：

左肺尖可见微结节影，边界较清。左肺上叶舌段可见小淡片影，边界欠清。双肺上叶可见小肺大泡影。余两侧肺野透过度良好，两肺纹理增强，两侧肺门形态、位置正常。主动脉壁及冠状动脉壁可见钙化，心脏大小正常。纵隔内未见肿大淋巴结。未见胸腔积液征像。双侧胸膜略增厚。

附带：双侧肩关节形态不整，密度不均。

诊断：

科内会诊意见：

1、左肺尖微结节，请随诊观察；
2、左肺上叶舌段少量炎性病变；
3、双肺上叶小肺大泡；
4、动脉硬化症；
5、双侧胸膜略增厚；
6、附带：双侧肩关节改变，请结合临床病史。

检查技师：　　报告医师：[redacted]　　审核医师：[redacted]

报告时间：2014-05-30 13:30:56

大连市中心医院放射科
DR诊断报告单

辽宁省医学影像检查结果互认单位　　ZXYY/CX-11-JL-23

患者类型：住院　　患者编号：[redacted]　　检查号[redacted]

姓　名：[redacted]　　性　别：女　　年　龄：59 岁

科　别：神经内科一病)　　设备型号：SHIMADZU DR

检查部位：[胸部后前位+右侧位]

胸部后前位及右侧位片：

胸廓完整对称，双侧肩关节间隙变窄，关节面伴增生硬化。两肺纹理增强，两肺内未见病灶。双侧肺门影不大。

纵隔不宽，心脏不大，主动脉迂曲。

两侧膈肌光滑，位置对称，两侧肋膈角锐利。

腰椎椎体见骨增生。

诊断：

胸部后前位及右侧位片：

1、肺纹理增强，主动脉迂曲。
2、双肩改变，请结合病史。
3、腰椎骨增生。

[redacted]

检查技师：　　报告医师：[redacted]　　审核医师：

报告日期：2014-05-19 15:26:14

OTOsuite

[redacted]
年龄:
出生日期:
报告日期: 2014/5/19
测试者:

大连市中心医院

电测听报告单

注：该检测结果仅反映受试者检测当时之情况。

气导: 贴耳式耳机, 骨导: B71 [贴耳式耳机]

右耳 (2014/5/19)

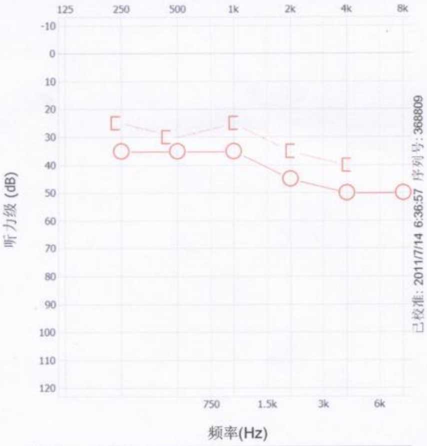

AC					
BC	0	0	0	0	0

气导: 贴耳式耳机, 骨导: B71 [贴耳式耳机]

左耳 (2014/5/19)

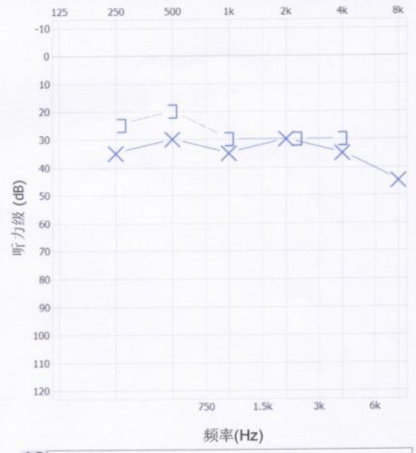

AC					
BC	0	0	0	0	0

Multi Weber

250	500	750	1k	1,5k	2k	3k	4k

纯音听阈均值(dB HL)/清晰度指数(%)

	AC	BC	AI
右	38	30	
左	31	26	

签名人:

图例

左		右		已掩蔽	
×		○	气导	□	△
>	^	<	骨导	]	[
S	S	S	声场	⊠	⊘
M		M	舒适阈		
U		U	不舒适阈		
↙	↓	↘	无响应		

纯音测听: 气导: 500, 1k, 2k; 骨导: 500, 1k, 2k

测听方法:

大连市中心医院心电图报告单

病区:神经内科一病房　床[redacted]

检查日期:2014-05-13 14:42:13

姓名[redacted]　ID [redacted]
性别:女　住院[redacted]
年龄:60岁

心率:76bpm
QRS:98ms
P-R:124ms

QT/QTc:372/402ms
QRS电轴:+64°
RV5/SV1:1.58/0.50mV

报告时间:2014-05-13 15:00:42
申请医生[redacted]

纸速: 25mm/s 灵敏度: 10mm/mv BL:ON AC:ON MF:OFF

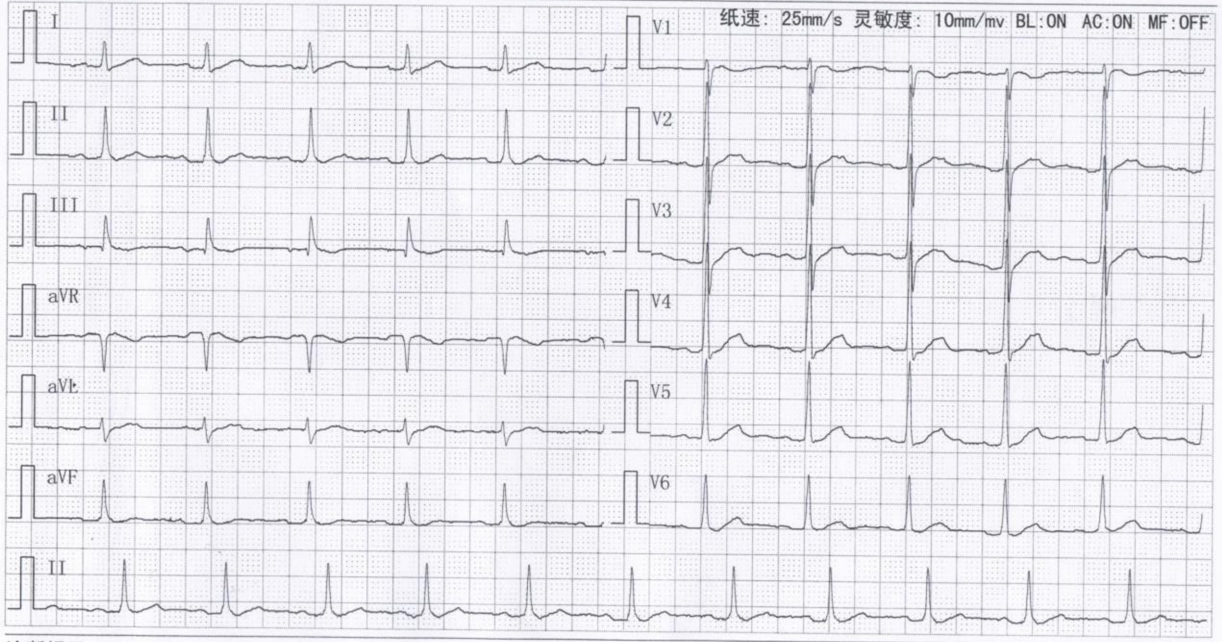

诊断提示:
　窦性心律
　心电轴不偏
　大致正常心电图

签名[redacted]

本报告仅供临床医生参考，报告医生签字有效

姓名：[redacted] 住院号码：[redacted] 样本种类：静脉血 样本编号：250
性别：女 科 室：内分泌科病房 、 采样日期：2014.05.26 0·样本状态：正常
年龄：60 岁 病人床号：[redacted] 送检医生：[redacted] 备注信息：

No	项 目	结 果		参 考 区 间	单位
1	游离T3	5.18		3.50-6.50	pmol/L
2	游离T4	16.93		11.50-22.70	pmol/L
3	促甲状腺激素	2.09		0.51-4.94	mIU/L
4	抗甲状腺球蛋白抗体	71.82	↑	0-60	U/ml
5	抗甲状腺过氧化物酶抗体	<28.00		0-60	U/ml

此线以下无检验项目

日期：14.05.26 08:20 报告日期：14.05.26 09:57 检验医生：[redacted] 审核医生[redacted]

此结果仅对所检测样本负责，样本保留72小时，如有疑问请及时与检验科联系。

[redacted] 住院号码: [redacted] 样本种类: 静脉血 样本编号: 10

女 科 室: 神经内科一病房 采样日期: 2014.05.14 05 样本状态: 正常

60 岁 病人床号: [redacted] 送检医生: [redacted] 备注信息:

项 目	结 果	参考区间	单位
糖化血红蛋白	5.8	4.0-6.5	%

此线以下无检验项目

期: 14.05.14 08:37 报告日期: 14.05.14 10:12 检验医生: [redacted] 审核医生: [redacted]

此结果仅对所检测样本负责，样本保留72小时，如有疑问请及时与检验科联系。

姓名：[redacted] 住院号码[redacted] 样本种类：静脉血 样本编号：113

性别：女 科 室：内分泌科病房 采样日期：2014.05.26 0 样本状态：正常

年龄：60 岁 病人床号：0546 送检医生：[redacted] 备注信息：

No	项 目	结 果		参 考 区 间	单位
1	黄体生成素	13.01		卵泡期：2.4-12.6；排卵期：14-95；黄体期：1.0-11.4；绝经期：7.7-58.5	mIU/ml
2	促卵泡刺激素	33.35		卵泡期：3.5-12.5；排卵期：4.7-21.5；黄体期：1.7-7.7；绝经期：25.8-134.8	mIU/ml
3	睾酮	0.11	↓	0.22-2.9	nmol/l
4	孕酮	0.97		卵泡期0.6-4.7排卵期2.4-9.4黄体期5.3-86绝经期0.3-2.5	nmol/l
5	雌二醇	25.29		卵泡90.1-716排卵期243-1509黄体期147-960绝经36.7-145	pmol/l
6	泌乳素	16.81		3.4-24.1	ng/ml

此线以下无检验项目

日期：14.05.26 08:35 报告日期：14.05.27 11:43 检验医生：[redacted] 审核医生：[redacted]

此结果仅对所检测样本负责，样本保留72小时，如有疑问请及时与检验科联系。

条码号：1403556242

病人姓名：[redacted]	住院号码：[redacted]	样本种类：血清	样本编号：3
病人性别：女	病人科室：内分泌科病房	采样日期：2014.05.27 10	样本状态：正常
病人年龄：60 岁	病人床号：[redacted]	送检医生：[redacted]	备注信息：

No	项 目	结 果	参 考 区 间	单位
1	生长激素	<0.05	<10.00	ng/ml

此线以下无检验项目

日期：14.05.27 10:10 报告日期：14.05.27 11:30 检验医生[redacted] 审核医生：[redacted]

此结果仅对所检测样本负责，样本保留72小时，如有疑问[redacted]科联系。

14.05.[illegible]

姓名：[redacted] 住院号码：[redacted] 样本种类：静脉血 样本编号：510

性别：女 科　室：内分泌科病房 采样日期：14.05.26 08 样本状态：正常

年龄：60 岁 床　号：[redacted] 送检医生：[redacted] 备注信息：

No	项　目	结　果	参考区间	单位	No	项　目	结　果	参考区间	单位
	餐后半小时血糖	7.32		mmol/L					

此线以下无检验项目

日期：14.05.26 09:41 报告日期：14.05.26 10:27 检验者：[redacted] 审核者：[redacted]

此结果仅对所检测样本负责，样本保留72小时，如有疑问请及时与检验科联系。

姓名：[redacted] 住院号码：[redacted] 样本种类：静脉血 样本编号：32
性别：女 科 室：内分泌科病房 采样日期：2014.05.26 0[illegible]样本状态：正常
年龄：60 岁 病人床号：[redacted] 送检医生：[redacted] 备注信息：

No	项 目	结 果	参考区间	单位
1	餐后半小时胰岛素	25.5		mU/L
2	餐后半小时C肽	2.02		ng/ml

此线以下无检验项目

[illegible]日期：14.05.26 09:54 报告日期：14.05.26 10:49 检验医生：[redacted] 审核医生：[redacted]

此结果仅对所检测样本负责，样本保留72小时，如有疑问请及时与检验科联系。

ZXYY/YL/ZY－020－JL－01

住院病人疾病诊断书 №0159475

姓名[redacted] 年龄 61 性别：男、女

单位

住院科室 内分泌 住院号 456682

住院日期自2016 年 5 月13 日至2016 年 6月 6 日

诊断：[illegible]

医师意见：

[illegible]

[illegible]

2型糖尿病

[illegible]

[illegible]

发给日期2016 年 6 月 6 日

经治医师 [redacted]

大 连 市 中 心 医 院

大连市中心医院
肌电诱发电位检查报告

ZXYY/YJ/ZY/DSL-007-JL-01

Study ID:
Name: [redacted]

Data of study: 2014-05-19
Age: 59

Patient: N1
Sex: 女性

脑干听觉诱发电位 BAEP 1ch AMP-3

Individual Data

			Lat		
			Left	Right	Side Diff
			ms	ms	ms
平均	Cz-A1 / Cz-A2	I	1.33	1.34	0.010
		II	2.3	2.2	0.10
		III	3.3	3.3	0
		IV	4.7	4.7	0
		V	5.4	5.3	0.10

Latency Difference

		Lat		
		ms	ms	ms
平均	Cz-A2:I - Cz-A2:III	1.97	1.96	0.010
	Cz-A2:III - Cz-A2:V	2.1	2.0	0.10
	Cz-A2:I - Cz-A2:V	4.1	4.0	0.10

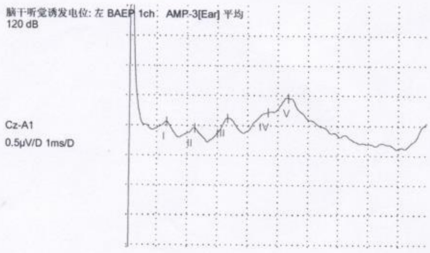

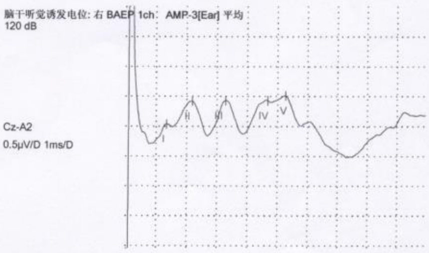

结论

正常 BAEP。

中心医院 L263 郭欣

[redacted] 住院号码：[redacted] 样本种类：静脉血 样本编号：2
性别：女 科 室：神经内科一病房 采样日期：2014.05.16 0[illegible] 样本状态：正常
年龄：60 岁 病人床号：[redacted] 送检医生：[redacted] 备注信息：

No	项 目	结 果	参考区间	单位
1	全段甲状旁腺激素	64.1	14.0-72.0	pg/ml

此线以下无检验项目

[illegible]验日期：14.05.16 08:41 报告日期：14.05.16 09:53 检验医生：[redacted] 审核医生：[redacted]

此结果仅对所检测样本负责，样本保留72小时，如有疑问请及时与检验科联系。

姓名[redacted] 住院号码[redacted] 样本种类：静脉血 样本编号：5
性别：女 科 室：神经内科一病房 采样日期：2014.05.20 0 样本状态：正常
年龄：60 岁 病人床号[redacted] 送检医生：[redacted] 备注信息：

No	项 目	结 果	参考区间	单位
1	全段甲状旁腺激素	71.0	14.0-72.0	pg/ml

此线以下无检验项目

检验日期：14.05.20 09:14 报告日期：14.05.20 10:06 检验医生：[redacted] 审核医生[redacted]

此结果仅对所检测样本负责，样本保留72小时，如有疑问请及时与检验科联系。

14.05.[illegible]

姓名：[redacted] 住院号码：[redacted] 样本种类：静脉血 样本编号：7
性别：女 科 室：内分泌科病房 采样日期：2014.05.26 0[illegible] 样本状态：正常
年龄：60 岁 病人床号[redacted] 送检医生：[redacted] 备注信息：

No	项 目	结 果	参考区间	单位
1	全段甲状旁腺激素	58.2	14.0-72.0	pg/ml

此线以下无检验项目

日期：14.05.26 09:52 报告日期：14.05.26 10:30 检验医生[redacted] 审核医生[redacted]

此结果仅对所检测样本负责，样本保留72小时，如有疑问请及时与检验科联系。

大 连 市 中 心 医 院

出院记录

姓名[redacted] 科别 内分泌科病房 床号[redacted] 住院号[redacted]

姓名[redacted] 性别 女 年龄 59岁 婚姻 已婚 住院号[redacted]

入院日期：2014.05.13 12:55:10 出院日期：2014.06.06

入院时情况（简要病史、阳性体征、有关实验室及器械检查结果）：患者以“头晕1天”入神经内科。该患者1天前无明显诱因开始突发出现头晕，感自身晃动，视物旋转，持续数秒，不敢睁眼，伴恶心及呕吐，右耳耳鸣，无发热，无咳嗽及咳痰，无抽搐，无意识障碍。既往：糖尿病史。入院查体：T：36.4℃ P：83次/分 R：18次/分 BP：116/70mmHg，心肺腹查体未及异常。神清，言语流利，颈部未闻及血管杂音，粗测视力正常，视野正常，双侧瞳孔等大等圆，直径2.5毫米，光反射灵敏，眼球运动正常，无眼震，咀嚼有力，额纹对称，鼻唇沟对称，面部感觉无异常，悬雍垂居中，转颈力可、耸肩有力，伸舌不偏。四肢肌力Ⅴ级，肌张力适中，肢体共济运动正常，步态不稳。深浅感觉正常。四肢腱反射对称引出。Babinski征双侧阴性。辅助检查：2014.05.12我院急诊头部CT提示未见异常，双侧眼球多发点状钙化。

入院诊断：周围性眩晕

2型糖尿病

低钾血症 高钙低磷原因待查

诊治经过：心电图：窦性心律，心电轴不偏，大致正常心电图。超声[心脏 结构 功能]：静息状态下，心内结构及血流未见明显异常，左室舒张功能减低。超声[颈动脉]：双侧颈动脉内膜增厚伴斑块形成。视频眼震电检查提示双侧水平半规管功能对称，位置试验（-）。BAEP：正常。TCD检查提示左侧椎动脉血流改变，注意该侧起始部狭窄可能，余颅内外动脉血流未见明显异常。胸部后前位及右侧位片：1、肺纹理增强，主动脉迂曲。2、双肩改变（双侧肩关节间隙变窄，关节面伴增生硬化）。3、腰椎骨增生。入院后给予抗血小板聚集，稳定动脉斑块，改善循环，改善脑供血等治疗，患者头晕症状逐渐好转。理化检查：血钾：2.73mmol/L，给予补钾治疗。总钙：3.08mmol/L，偏高，无机磷：0.75mmol/L，镁：0.52mmol/L，低，因离子紊乱，转入内分泌科。血总钙：2.87-3.08mmol/L均高，无机磷：0.75-0.85mmol/L，镁：0.39-0.49mmol/L低；①24小时尿总钙定量：2.13mmol/24h，24小时尿无机磷定量：15.1mmol/24h。②24小时尿肌酐定量：3.52mmol/24h，24小时尿钾定量：27.8mmol/24h（同步血钾：2.80mmol/L）；，24小时尿钠定量：127.5mmol/24h，24小时尿氯化物定量：113.8mmol/24h，24小时尿总钙定量：1.37mmol/24h，24小时尿无机磷定量：9.3mmol/24h；③24小时尿肌酐定量：7.48mmol/24h，24小时尿尿酸定量：3.09mmol/24h，24小时尿钾定量：77.8mmol/24h（同步血钾：2.98mmol/L），24小时尿钠定量：194.4mmol/24h，24小时尿氯化物定量：210.6mmol/24h，24小时尿总钙定量：1.66mmol/24h，24小时尿无机磷定量：20.2mmol/24h，24小时尿葡萄糖定量：0.04mmol/24h。提示高血钙，低尿钙，低血钾、低血镁，高尿钾；完善PTH检查均正常（全段甲状旁腺激素①：64.1pg/ml②71.0pg/ml；③58.2pg/ml）。超声[甲状腺及淋巴结]：1.双侧甲状腺多发囊性小结节，建议定期复查。2.甲状腺左叶上极背侧后方低回声结节，不除外甲状旁腺病变。外院行甲状旁腺MIBI显像检查未见异常。不支持原发性甲状旁腺功能亢进。完善相关检查以除外是否存在肿瘤疾病所致血钙高。CT[双肺，平扫]：1、左肺尖微结节，请随诊观察；2、左肺上叶舌段少量炎性病变；3、双肺上叶小肺大泡；4、动脉硬化症；5、双侧胸膜略增厚；6、附带：双侧肩关节改变。

大 连 市 中 心 医 院

出院记录

姓名[redacted] 科别 内分泌科病房 床[redacted] 住院[redacted]

超声[肝 胆 脾 胰 双肾]:肝胆脾胰双肾未见明显异常。CT[甲状腺,平扫]:甲状腺肿大;胸廓入口区伪影较重,两侧叶密度显示欠均匀;左叶下极处隐约小低密度灶。超声:双肾输尿管膀胱未见明显占位性病变;左侧附件区囊肿（子宫全切除术后,双侧卵巢未显示,左侧附件区见大小约32x20x20mm囊肿,形态规则,边界清晰,内呈液性,其内及周边未见明显血流信号,盆腔未见明显积液影像）,嘱复查随诊。超声:双侧乳腺增生并局部导管扩张 左侧乳腺实性占位(BI-RADS 3级)（双侧乳腺腺体稍厚,层次结构紊乱,内呈网格状回声,分布不均,左侧乳腺局部导管扩张,内径约0.23cm,右侧乳腺局部导管扩张,内径约0.25cm。左侧乳腺可探及实性结节,位于外象限3点,大小约0.55×0.45cm,距乳头约2.0cm,距体表约0.64cm,边界清晰,形态规整,后方回声不变。CDFI:内部及周边未见血流信号。双侧腋窝探查,未见明显肿大淋巴结）。请外科会诊,建议3个月复查,外科随诊。既往胃镜检查未见异常。血甲胎蛋白、癌胚抗原、糖类抗原19-9、糖类抗原12-5、糖类抗原15-3均正常。目前无恶性肿瘤所致血钙增高依据。甲功及抗甲状腺过氧化物酶抗体正常。抗甲状腺球蛋白抗体：71.82U/ml略高。泌乳素、生长激素、性腺系列、皮质醇均正常。血压正常,无阵发性高血压、心悸等表现。血清K轻链、血清L轻链均正常。分析患者高血钙 低尿钙为家族性低尿钙性高钙血症可能性大,明确需行基因检测。此外患者低钾、低镁,24小时尿钾高,提示有肾性失钾,患者无高血压,血醛固酮正常（醛固酮：130pg/ml,血管紧张素II：40pg/ml;均正常）。尿常规PH7.0-8.0,三次尿比重均为：1.010;查血渗透压288mOsm/kg,同步查尿渗透压849mOsm/kg,无尿渗透压下降。血气血液酸碱度：7.404,动脉二氧化碳分压：44.0mmHg,动脉血氧分压：77.8mmHg,实际碳酸氢盐浓度：26.9mmol/L,标准碳酸氢盐浓度：26.0mmol/L,缓冲碱：1.8mmol/L。患者既往发现高钙病史久,血钾情况不详,血钾低不除外高钙所致肾间质损伤。肾内科会诊低血钾考虑需除外Gitelman综合征可能,建议完善基因检测。糖化血红蛋白：5.8%,示近2、3个月平均血糖控制佳。监测血糖显示,暂不需药物。血常规、肝功（碱性磷酸酶：48U/L;低于正常）、肾功（尿素：2.71mmol/L,肌酐：34umol/L）、血脂（总胆固醇：3.78mmol/L,三酰甘油：0.78mmol/L,高密度脂蛋白胆固醇：1.19mmol/L,低密度脂蛋白胆固醇：1.88mmol/L）大致正常。患者长期口服他汀药物稳定斑块,继续该药口服。眼科会诊动脉硬化I期,屈光间质混浊,网膜可见萎缩灶及渗出,未见明显出血。尿微量白蛋白：0.49mg/dl;肌酐：3495umol/L;计算白蛋白/肌酐12.4mg/g,暂无早期糖尿病肾病依据。骨密度：腰椎骨量减少（T:-1.5～-0.5;Z：0.9-2.0）;右侧股骨骨量减少（T:-2.0～ -0.5;Z:-0.1～1.1）。

出院情况：患者无抽搐,无发热,无乏力,无头晕,无恶心呕吐,血压110/70mmHg,血糖5.6mmol/l左右,餐后血糖6-10mmol/l。钾：3.52mmol/L,钠：140.1mmol/L,氯化物：103.1mmol/L,碳酸氢根：24.1mmol/L,总钙：2.88mmol/L,无机磷：0.76mmol/L,镁：0.39mmol/L。

出院诊断：高钙血症原因待查 家族性低尿钙性高钙血症可能性大
低钾血症 低镁血症
周围性眩晕
2型糖尿病
双侧颈动脉内膜增厚伴斑块形成

出院医嘱：1.避免脱水,规律饮食、运动
2.氯化钾缓释片1.5g,日3次口服（依据血钾调整用量）

氢苯噻嗪 1粒 [illegible]

ZXYY/CX-07-JL-23

大 连 市 中 心 医 院

出院记录

姓名[redacted] 科别 内分泌科病房 床号[redacted] 住院号 [redacted]

门冬氨酸钾镁片 2粒，日3次口服 （监测血镁，调整用量）

阿司匹林肠溶片[合资] 100mg，日1次口服（如有出血性疾病停用）

阿托伐他汀钙片 10mg，日1次晚睡前口服（注意复查血脂 肝功酶 肌酶）

3. 内分泌科复查随诊

监测血糖，如血糖不稳定，门诊就诊，必要时药物治疗

监测血离子，门诊就诊，调整用药

针对双侧乳腺增生并局部导管扩张 左侧乳腺实性占位(BI-RADS 3级)建议3个月复查，外科随诊

4. 病情变化立即就诊

门诊预约挂号： 114 www.dlzxyy.com 84440555 84442555

主治医师[redacted]/住院医师[redacted]

姓名[redacted] 住院号码[redacted] 样本种类：静脉血 样本编号：31
性别：女 科 室：内分泌科病房 采样日期：2014.05.26 0·样本状态：正常
年龄：60 岁 病人床号[redacted] 送检医生：[redacted] 备注信息：

No	项 目	结 果	参考区间	单位
1	胰岛素	11.1	3.0-25.0	mU/L
2	C肽	1.18	0.81-3.85	ng/ml

此线以下无检验项目

[illegible]日期：14.05.26 09:54 报告日期：14.05.26 10:49 检验医生：[redacted] 审核医生[redacted]

此结果仅对所检测样本负责，样本保留72小时，如有疑问请及时与检验科联系。

[illegible]

姓名：[redacted] 住院号码：[redacted] 样本种类：静脉血 样本编号：147

性别：女 科 室：神经内科一病房 采样日期：14.05.14 05 样本状态：正常

年龄：60 岁 床 号：[redacted] 送检医生：[redacted] 备注信息：

No	项 目	结 果		参考区间	单位
1	白细胞计数	5.30		3.5 9.5	*10^9/L
2	中性粒细胞百分数	55.8		40-75	%
3	淋巴细胞百分数	35.7		20-50	%
4	单核细胞百分数	7.0		3.0-10.0	%
5	嗜酸粒细胞百分数	1.1		0.4-8.0	%
6	嗜碱粒细胞百分数	0.4		0-1.0	%
7	中性细胞绝对值	3.0		1.80-6.30	*10^9/L
8	淋巴细胞绝对值	1.9		1.1-3.2	*10^9/L
9	单核细胞绝对值	0.4		0.1 0.6	*10^9/L
10	嗜酸粒细胞绝对值	0.06		0.02-0.52	*10^9/L
11	嗜碱粒细胞绝对值	0.02		0-0.06	*10^9/L
12	红细胞计数	3.97		3.8-5.1	*10^12/L
13	血红蛋白	132		115-150	G/L
14	红细胞压积	36.4		35.0-45.0	%
15	平均红细胞体积	91.7		82.0-100.0	FL
16	平均血红蛋白量	33.2		27.0-34.0	PG
17	平均血红蛋白浓度	363.0	↑	316 354	G/L
18	RDW-CV	11.8			%
19	RDW-SD	39.8		37-50	fl
20	血小板计数	225		125-350	*10^9/L
21	血小板压积	0.25			%
22	血小板平均体积	10.9		9.0-13.0	FL
23	血小板分布宽度	13.1		9.0-17.0	%

此线以下无检验项目

检验日期：14.05.14 00:00 报告日期：14.05.14 09:47 检验者：[redacted] 审核者：[redacted]

此结果仅对所检测样本负责，样本保留72小时，如有疑问请及时与检验科联系。

病人姓名：[redacted] 住院号码[redacted] 样本种类：静脉血 样本编号：20
病人性别：女 病人科室：内分泌科病房 采样日期：2014.05.26 04 样本状态：正常
病人年龄：60 岁 病人床号：[redacted] 送检医生：[redacted] 备注信息：

No	项 目	结 果	参 考 区 间	单位
1	血清K轻链	1267	629-1350	mg/dl
2	血清L轻链	645	313-723	mg/dl

此线以下无检验项目

日期：14.05.26 08:42 报告日期：14.05.26 09:41 检验医生：[redacted] 审核医生：[redacted]

此结果仅对所检测样本负责，样本保留72小时，如有疑问请及时与检验科联系。

大连市中心医院放射科
CT诊断报告单

ZXYY/CX-11-JL-27　　辽宁省医学影像检查结果互认单位

患者类型：住院　　**患者编号**[redacted]　　**检查号**[redacted]

姓名：[redacted]　　**性别：**女　　**年龄：** 59

科　别： 内分泌科病房　　**设备：**GE CT64

检查部位：[甲状腺,平扫]

甲状腺MSCT平扫检查所见:

甲状腺进入胸廓入口；胸廓入口区伪影较重，两侧叶密度显示欠均匀，左叶下极处隐约可见小低密度灶，边界模糊，直径约0.6cm。颈部未见确切肿大淋巴结。周围软组织结构未见异常。

诊断：

甲状腺肿大；胸廓入口区伪影较重，两侧叶密度显示欠均匀；左叶下极处隐约小低密度灶；建议结合超声检查。

检查技师：　　**报告医师：**[redacted]　　**审核医师：**[redacted]

报告时间：2014-05-30 11:29:08

大连市中心医院 ZXYY/YL/ZY/SJNY/039-JL-01

经颅多普勒超声（TCD）诊断报告

姓名：[redacted]　　性别：女　　年龄：60 岁　　申请科室：神内一科

病历号：[redacted]　　检查日期:20140514　　仪器型号：EMS

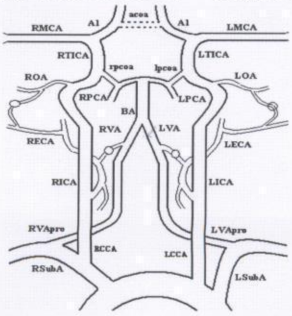

超声所见：双侧颈总动脉颈内动脉锁骨下动脉血流速度正常，频谱形态正常，血流声频未闻异常，血流搏动指数正常。

双侧大脑中动脉前动脉后动脉颈内动脉终末段血流速度正常，频谱形态正常，血流声频未闻异常，血流搏动指数正常。

双侧椎动脉血流不对称，左侧血流速度减慢，搏动指数降低，声频正常。右侧椎动脉及基底动脉血流速度正常，频谱形态正常，血流声频未闻异常，血流搏动指数正常。

超声提示：左侧椎动脉血流改变，注意该侧起始部狭窄可能，余颅内外动脉血流未见明显异常。

[redacted]

姓名[redacted] 住院号码[redacted] 样本种类：静脉血 样本编号：120

性别：女 科 室：内分泌科病房 采样日期：2014.05.26 0 样本状态：正常

年龄：60 岁 病人床号：[redacted] 送检医生：[redacted] 备注信息：

No	项 目	结 果	参考区间	单位
1	甲胎蛋白	2.76	0-7.0	ng/ml
2	癌胚抗原	0.69	0-3.4	ng/ml
3	糖类抗原19-9	13.64	0-39.0	U/ml
4	糖类抗原12-5	6.80	0-35.0	U/ml
5	糖类抗原15-3	3.17	0-25	U/ml

此线以下无检验项目

[redacted]

[illegible]验日期：14.05.26 08:35 报告日期：14.05.26 10:46 检验医生：[redacted] 审核医生：

此结果仅对所检测样本负责，样本保留72小时，如有疑问请及时与检验科联系。

大连市中心医院

超声检查切面显像（彩色）报告单

ZXYY/CX-11-JL-28

住院号:[redacted]　　姓　名:[redacted]　　性　别:女　　年　龄:59　岁

临床诊断:

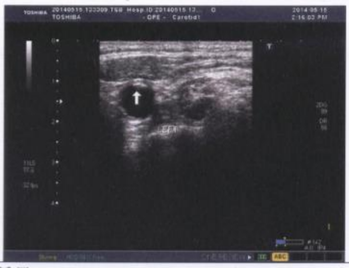
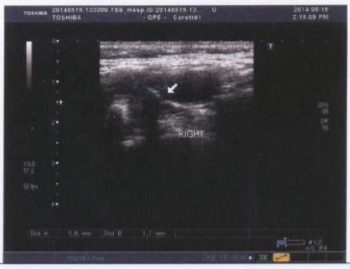

检查所见:

	D（mm）	IMT（mm）	PSV(cm/s)		D（mm）	IMT（mm）	PSV(cm/s)
RCCA	6.1	1.1	52	LCCA	7.2	1.1	62
R-Bulb	7.0	1.0	46	L-Bulb	6.6	1.0	53
RICA	5.3		62	LICA	5.1		69
RECA			68	LECA			57
RVA	3.3		44	LVA	3.6		52
RSCA			127	LSCA			104

检查所见:

双侧颈总动脉管径对称，内膜增厚，右侧分叉处右后壁可见大小约5.8x1.7mm不均回声扁平斑，左侧分叉处后壁及前壁分别可见大小约5.1x1.8mm、4.7x1.5mm不均回声扁平斑，双侧流速正常。

双侧颈动脉球部管径对称，内膜增厚，流速正常。

双侧颈内动脉管径对称，各段血流速度正常。

双侧椎动脉管径对称，流速正常。

双侧颈外动脉及锁骨下动脉血流速度未见明显异常。

诊断:

双侧颈动脉内膜增厚伴斑块形成

报告医师:[redacted]

报告时间:2014-05-15

医师签字:[redacted]

大连市中心医院

超声检查切面显像（彩色）报告单

ZXYY/CX-11-JL-28

住院号:[redacted]　　姓　名[redacted]　　性　别:女　　年　龄:59　岁

临床诊断:

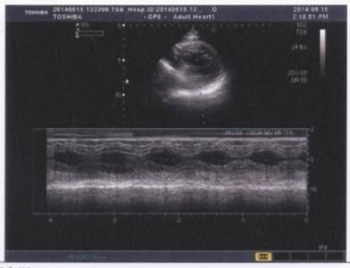

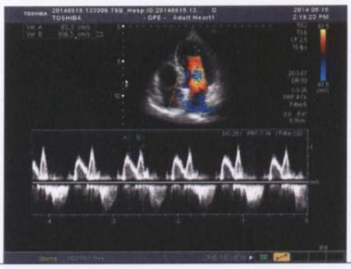

检查所见:

RVOT 24mm, AOD 25mm, LAD 33mm, RVD 18mm, IVS 9mm, LVD 42mm, LVPW 8mm. MPA 20mm, AAO 36mm. 心尖四腔心切面观:LA 37x47mm, RA 35x42mm.
主动脉血流速度 1.1m/s; 肺动脉血流速度 1.0m/s。
心脏结构:
1. 主动脉搏动尚可,主波重搏波存在。
2. 各房室腔内径正常范围。室间隔及左、右室壁厚度正常，运动协调，收缩幅度正常,室壁运动未见明显节段性异常。
3. 各瓣膜形态、结构、启闭运动未见异常。大动脉关系、内径正常。心包腔未见异常。
多普勒检查：二、三尖瓣房侧收缩期均可见微少量返流信号。
心功能测定:
E:0.8m/s, A:1.1m/s, E/A<1, EF 66%, FS 36%, SV 67ml, IRT 102ms.

诊断:
静息状态下
心内结构及血流未见明显异常
左室舒张功能减低

报告医师[redacted]
报告时间:2014-05-15
医师签字:[redacted]

大连市中心医院

超声检查切面显像（彩色）报告单

ZXYY/CX-11-JL-28

住院号[redacted]　　姓　名[redacted]　　性　别:女　　年　龄:59　岁

临床诊断:

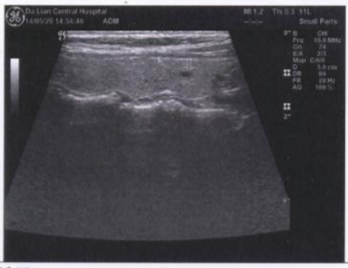

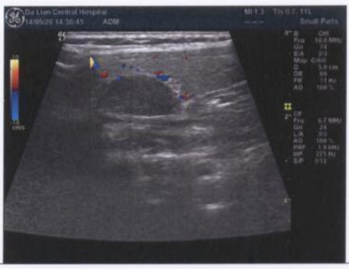

检查所见:

甲状腺位置正常，左叶大小约4.5x1.8x1.7cm，右叶大小约4.5x1.9x1.8cm，峡部厚约0.3cm。双侧甲状腺均可见多个结节，左叶较大者约0.3x0.2cm，右叶较大者约0.3x0.2cm，形态尚规整，边界较清晰，内呈无回声，其内部及周边未见血流信号，峡部未见明显结节影像。

另于左侧上极背侧后方可见大小1.8x1.0cm低回声结节，形态尚规整，边界尚清晰，外周可见高回声包膜，与食管无明显关系，其周边可见少许血流信号。

双侧颈部未见明显肿大淋巴结。

诊断:

1. 双侧甲状腺多发囊性小结节，建议定期复查。
2. 甲状腺左叶上极背侧后方低回声结节，
   不除外甲状旁腺病变，请结合临床。

报告医师[redacted]

报告时间:2014-05-20

医师签字[redacted]

大连市中心医院

超声检查切面显像（彩色）报告单

ZXYY/CX-11-JL-28

住院号[redacted]　　姓　名[redacted]　　性　别:女　　年　龄:59　岁

临床诊断：

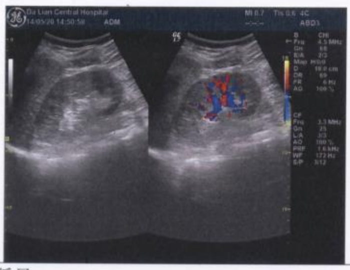

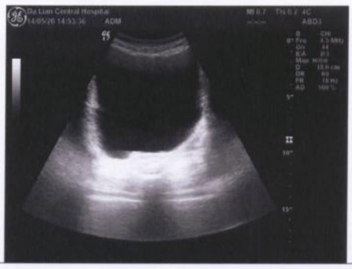

检查所见：

左肾大小约10.4x4.6cm，实质部厚约1.7cm， 右肾大小约9.6x4.6cm，实质部厚约1.5cm。双肾形态规整，皮髓质界限清晰,集合系统未见分离，肾区未见明显占位性病变。
双侧输尿管未见明显扩张。
膀胱充盈尚可，其内未见明显异常回声。

诊断：
双肾输尿管膀胱未见明显占位性病变。

报告[redacted]
报告[redacted]
医师[redacted]

姓名：[redacted] 住院号码：[redacted] 样本种类：尿 样本编号：484
性别：女 科 室：内分泌科病房 采样日期：14.05.26 06 样本状态：正常
年龄：60 岁 床 号：0546 送检医生：[redacted] 备注信息：

No 项 目	结 果	参考区间	单位	No 项 目	结 果	参考区间	单位
肌酐	3495		umol/L				

此线以下无检验项目

日期：14.05.26 09:39 报告日期：14.05.26 10:24 检验者：[redacted] 审核者[redacted]

此结果仅对所检测样本负责，样本保留72小时，如有疑问请及时与检验科联系。

病人姓名：[redacted] 住院号码：[redacted] 样本种类：尿 样本编号：14
病人性别：女 病人科室：内分泌科病房 采样日期：2014.05.26 06 样本状态：正常
病人年龄：60 岁 病人床号：[redacted] 送检医生：[redacted] 备注信息：

No	项 目	结 果	参 考 区 间	单位
1	尿微量白蛋白	0.49	0-1.90	mg/dl

此线以下无检验项目

日期：14.05.26 08:50 报告日期：14.05.26 09:55 检验医生：[redacted] 审核医生：[redacted]

此结果仅对所检测样本负责，样本保留72小时，如有疑问请及时与检验科联系。

4.05.27

第 页

姓名: [redacted] 住院号码: [redacted] 样本种类: 尿 样本编号: [illegible]

性别: 女 科 室: 神经内科一病房 采样日期: 14.05.14 06 样本状态: 正常

年龄: 60 岁 床 号: [redacted] 送检医生: [redacted] 备注信息:

No	项 目	结 果	参考区间	单位
1	颜色	淡黄		
2	尿糖	norm	-	mmol/L
3	尿蛋白	neg	-	g/L
4	尿胆红素	neg	-	umol/l
5	尿胆原	norm	+-	umol/L
6	尿酮体	neg	-	mmol/L
7	潜血	neg	-	
8	尿酸碱度	7.0	5.4-8.4	
9	尿比重	1.010	1.003-1.03	
10	尿亚硝酸盐	neg		
11	白细胞	neg	-	
12	维生素C	neg	-	g/L
13	RBC	0.0	0-5	/ul
14	WBC	6.5	0-9	/ul
15	鳞状上皮细胞	2.3	0-5	/ul
16	非鳞状上皮细胞	0.0	0-2	/ul
17	结晶	1.3	0-6	/ul
18	病理管型	0.0	0-1.5	/ul
19	透明管型	0.0	0-2	/ul
20	细菌	19.8	0-75	/ul
21	酵母样细胞	0.0	0-3	/ul
22	粘液丝	38.3	0-528	/ul

此线以下无检验项目

检验日期: 14.05.14 08:07 报告日期: 14.05.14 08:49 检验者: [redacted] 审核者 [redacted]

此结果仅对所检测样本负责，样本保留72小时，如有疑问请及时与检验科联系。

姓名: [redacted] 住院号码: [redacted] 样本种类: 尿 样本编号: 45

性别: 女 科 室: 内分泌科病房 采样日期: 14.05.26 06 样本状态: 正常

年龄: 60 岁 床 号: [redacted] 送检医生: [redacted] 备注信息:

No	项 目	结 果		参考区间	单位
1	颜色	淡黄			
2	尿糖	norm		-	mmol/L
3	尿蛋白	neg		-	g/L
4	尿胆红素	neg		-	umol/l
5	尿胆原	norm		+-	umol/L
6	尿酮体	neg		-	mmol/L
7	潜血	neg		-	
8	尿酸碱度	8.0		5.4-8.4	
9	尿比重	1.010		1.003-1.03	
10	尿亚硝酸盐	neg		-	
11	白细胞	neg		-	
12	维生素C	neg		-	g/L
13	RBC	0.0		0-5	/ul
14	WBC	6.9		0-9	/ul
15	鳞状上皮细胞	5.2	↑	0-5	/ul
16	非鳞状上皮细胞	0.0		0-2	/ul
17	结晶	0.0		0-6	/ul
18	病理管型	0.0		0-1.5	/ul
19	透明管型	0.0		0-2	/ul
20	细菌	45.8		0-75	/ul

No	项 目	结 果	参考区间	单位
21	酵母样细胞	0.0	0-3	/ul
22	粘液丝	106.9	0-528	/ul

此线以下无检验项目

检验日期: 14.05.26 08:21 报告日期: 14.05.26 09:18 检验者: [redacted] 审核者: [redacted]

此结果仅对所检测样本负责，样本保留72小时，如有疑问请及时与检验科联系。

姓名：[redacted] 住院号码：[redacted] 样本种类：尿 样本编号：137
性别：女 科 室：内分泌科病房 采样日期：14.05.29 08 样本状态：正常
年龄：60 岁 床 号：[redacted] 送检医生：[redacted] 备注信息：

No	项 目	结 果		参考区间	单位
1	颜色	淡黄			
2	尿糖	norm		-	mmol/L
3	尿蛋白	neg		-	g/L
4	尿胆红素	neg		-	umol/l
5	尿胆原	norm		+-	umol/L
6	尿酮体	neg		-	mmol/L
7	潜血	neg		-	
8	尿酸碱度	8.0		5.4-8.4	
9	尿比重	1.010		1.003-1.03	
10	尿亚硝酸盐	neg		-	
11	白细胞	+ 25	↑	-	
12	维生素C	neg		-	g/L
13	RBC	5.0		0-5	/ul
14	WBC	9.3	↑	0-9	/ul
15	鳞状上皮细胞	5.3	↑	0-5	/ul
16	非鳞状上皮细胞	0.0		0-2	/ul
17	结晶	1.3		0-6	/ul
18	病理管型	0.0		0-1.5	/ul
19	透明管型	0.0		0-2	/ul
20	细菌	40.9		0-75	/ul
21	酵母样细胞	0.0		0-3	/ul
22	粘液丝	1522.0	↑	0-528	/ul

此线以下无检验项目

检验日期：14.05.29 08:20 报告日期：14.05.29 10:11 检验者：[redacted] 审核者[redacted]

此结果仅对所检测样本负责，样本保留72小时，如有疑问请及时与检验科联系。

大连市中心医院神经内科视频眼震电图检查报告

ZXYY/YL/ZY/SJNY-038-JY-01

姓名[redacted] 性别 女 年龄 61 病例号

一．眼动[redacted]

1．凝视检查

	水平		垂直	
30°	–	–	–	-
20°	–	–	–	–

2．扫视：正常/异常 ✓正常

3．视跟踪：I ✓ II III IV

4．视动眼震：对称性：对称

二．位置性眼震评价

1．自发性眼震：无

2．摇头眼震：/

3．位置眼震：

平卧	右侧卧	左侧卧	悬头	左侧悬头	右侧悬头
–	–	–	–	–	–

三．变位性眼震评价：Dix-Hallpike：阴性

四．前庭双温评价（冷热气）

		眼震方向	慢相角速度（° /S）	固视抑制
47°	右	左	6	[illegible]
	左	左	5	
20°	右			
	左			

R47+R20=　　L47+L20=　　CP(左，右)= 9 %　正常值（0-25%）

五．冰水试验：右耳左向眼震　左耳右向眼震　CP(左，右)=　%

最后印象：双侧水平半规管功能对称，位置试验[illegible]

检查者：[redacted] 检查日期：2014 年 5 月 16 日
